# *Nf1* mutation disrupts activity-dependent oligodendroglial plasticity and motor learning in mice

**DOI:** 10.1038/s41593-024-01654-y

**Published:** 2024-05-30

**Authors:** Yuan Pan, Jared D. Hysinger, Belgin Yalçın, James J. Lennon, Youkyeong Gloria Byun, Preethi Raghavan, Nicole F. Schindler, Corina Anastasaki, Jit Chatterjee, Lijun Ni, Haojun Xu, Karen Malacon, Samin M. Jahan, Alexis E. Ivec, Benjamin E. Aghoghovwia, Christopher W. Mount, Surya Nagaraja, Suzanne Scheaffer, Laura D. Attardi, David H. Gutmann, Michelle Monje

**Affiliations:** 1 Department of Neurology and Neurological Sciences, Stanford University, Stanford, CA USA; 2 Department of Symptom Research, University of Texas MD Anderson Cancer Center, Houston, TX USA; 3 Department of Neuro-Oncology, University of Texas MD Anderson Cancer Center, Houston, TX USA; 4 Howard Hughes Medical Institute, Stanford University, Stanford, CA USA; 5 Department of Neurology, Washington University School of Medicine, St. Louis, MO USA; 6 Department of Radiation Oncology, Stanford University, Stanford, CA USA; 7 Department of Genetics, Stanford University, Stanford, CA USA

**Keywords:** Oligodendrocyte, Developmental disorders

## Abstract

Neurogenetic disorders, such as neurofibromatosis type 1 (NF1), can cause cognitive and motor impairments, traditionally attributed to intrinsic neuronal defects such as disruption of synaptic function. Activity-regulated oligodendroglial plasticity also contributes to cognitive and motor functions by tuning neural circuit dynamics. However, the relevance of oligodendroglial plasticity to neurological dysfunction in NF1 is unclear. Here we explore the contribution of oligodendrocyte progenitor cells (OPCs) to pathological features of the NF1 syndrome in mice. Both male and female littermates (4–24 weeks of age) were used equally in this study. We demonstrate that mice with global or OPC-specific *Nf1* heterozygosity exhibit defects in activity-dependent oligodendrogenesis and harbor focal OPC hyperdensities with disrupted homeostatic OPC territorial boundaries. These OPC hyperdensities develop in a cell-intrinsic *Nf1* mutation-specific manner due to differential PI3K/AKT activation. OPC-specific *Nf1* loss impairs oligodendroglial differentiation and abrogates the normal oligodendroglial response to neuronal activity, leading to impaired motor learning performance. Collectively, these findings show that *Nf1* mutation delays oligodendroglial development and disrupts activity-dependent OPC function essential for normal motor learning in mice.

## Abstract (web summary)

Activity-dependent oligodendroglial plasticity contributes to neuronal functions. Here the authors show that adaptive oligodendrocyte progenitor cell responses are disrupted in neurofibromatosis 1, impairing oligodendroglial dynamics and resulting in motor learning deficits in *Nf1*-deficient and *Nf1*-mutant mice.

## Main

In the central nervous system (CNS), oligodendrocyte progenitor cells (OPCs) continuously proliferate throughout life, giving rise to oligodendrocytes, which are critical for myelination and the proper function of neural circuits^1–3^. In many brain regions, OPC division and differentiation are tightly regulated by neuronal activity, thus coupling adaptive myelination to neuronal signal propagation and network function^2,4–6^. As such, disruption of activity-regulated OPC differentiation results in wide-ranging effects on multiple domains of cognition, including attention, learning and memory^6–9^. For example, genetic inhibition of OPC differentiation results in motor learning deficits on the complex wheel test^7,8^.

While neuronal activity-dependent regulation of oligodendroglial dynamics is essential for normal CNS function, the contribution of dysregulated activity-dependent oligodendrogenesis to neurological and neuropsychiatric disorders is just beginning to come to light. Prior studies have shown that activity-regulated OPC proliferation, oligodendrogenesis and myelination are disrupted following chemotherapy, which contributes to chemotherapy-related cognitive impairment in mice^6^. Similarly, in rodent models of absence epilepsy, OPC proliferation, oligodendrocyte numbers and myelination are increased within the seizure network, and this aberrantly increased maladaptive myelination contributes to epilepsy progression such that genetic or pharmacological blockade of activity-regulated oligodendrogenesis decreases seizure frequency^10^. In another example of dysregulated oligodendroglial precursor proliferation leading to disease, OPCs can serve as a cell of origin for both low- and high-grade gliomas^11–14^.

The contribution of OPCs to both neurological dysfunction and gliomagenesis is particularly germane to neurofibromatosis type 1 (NF1), a cancer predisposition syndrome in which affected individuals are also prone to learning, behavioral and motor deficits. Patients with NF1 are born with a germline inactivating mutation in one copy of the *NF1* gene (monoallelic or heterozygous *NF1* loss) but may acquire a ‘second-hit’ mutation (biallelic *NF1* loss) during development in susceptible cell types to induce glioma formation^15,16^. In addition to increased brain tumor risk, children with NF1 exhibit impairments in attention, learning, working memory, executive function, motor function and motor learning^17–19^, which could reflect abnormalities in adaptive myelination^4,6,7,9^. Support for dysregulated OPC function in the setting of NF1 derives from several studies: analysis of heterozygous *Nf1*-mutant mice reveals increased OPC density in the spinal cord^20^, while *Nf1* genetic knockdown in zebrafish results in increased spinal cord OPC proliferation, density and migration^21^. Similarly, using the mosaic analysis with double markers (MADM) model, *Nf1*-null OPCs exhibit increased proliferation and decreased differentiation in vivo^22^. In this Article, we leveraged optogenetic and behavioral approaches coupled with numerous mouse strains harboring different NF1 patient germline *Nf1* gene mutations and OPC-specific *Nf1* loss to demonstrate that *Nf1* mutation in OPCs disrupts their adaptive responses, impairs oligodendroglial dynamics and results in motor learning deficits.

## Impaired *Nf1*-mutant OPC responses to neuronal activity

**Fig. 1:**
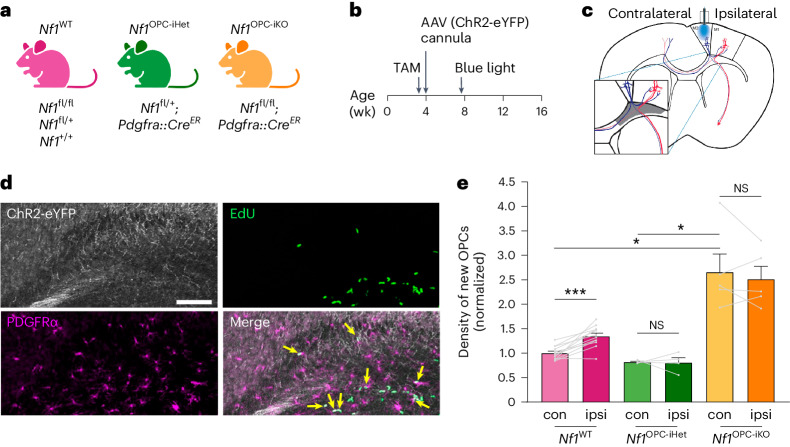
*Nf1*-mutant OPCs lack adaptive proliferative responses to increased neuronal activity. **a**, Mouse strains used. **b**, Experimental design. Tamoxifen (TAM) was given for 4 consecutive days starting at 3 weeks of age. AAV injection and cannula implantation into the premotor (M2) occurred at 4 weeks of age. Blue light stimulation at 7 weeks of age. **c**, Optogenetic stimulation of the ipsilateral side. Inset, the cingulum (gray), where M2 axons (blue) are concentrated. **d**, Representative IF images of the cingulum revealed cells expressing ChR2-eYFP (white), EdU (green) and PDGFRα (magenta). Arrows, proliferating OPCs (EdU^+^/PDGFRα^+^). Scale bar, 100 µm. **e**, Immunohistochemistry revealed an increased density of proliferating OPCs (EdU^+^/PDGFRα^+^) in the ipsilateral stimulated (ipsi, dark color) side, relative to the contralateral unstimulated (con, light color) side, in the brains of *Nf1*^WT^ mice (*N* = 12). No change between ipsilateral and contralateral sides was observed in *Nf1*^OPC-iHet^ (*N* = 4) and *Nf1*^OPC-iKO^ (*N* = 5) mice. ***P = 0.0008; *P = 0.041 (*Nf1*^WT^ contralateral versus *Nf1*^OPC-iKO^ contralateral), 0.0284 (*Nf1*^OPC-iHet^ contralateral versus *Nf1*^OPC-iKO^ contralateral). The density of new OPCs is normalized to contralateral *Nf1*^WT^ values in each cohort. Each point represents one mouse. Brown–Forsythe ANOVA test (*F* = 19.1) with Dunnett’s T3 multiple comparisons. Data shown as mean ± s.e.m.; two-sided; NS, not significant (*P* > 0.05). Source data

To determine how *Nf1* mutation might affect the adaptive responses of OPCs to neuronal activity, we generated mice that were genetically wild-type (WT)-equivalent (*Nf1*^WT^: *Nf1*^*+/+*^, *Nf1*^*fl/+*^ or *Nf1*^*fl/fl*^), OPC-specific heterozygous *Nf1*-mutant (*Nf1*^OPC-iHet^: *Nf1*^*fl/+*^*;Pdgfra::Cre*^*ER*^; i, inducible; tamoxifen injected at P24) and OPC-specific *Nf1*-null (*Nf1*^OPC-iKO^: *Nf1*^*fl/fl*^*;Pdgfra::Cre*^*ER*^, tamoxifen injected at P24) (Fig. 1a). Adeno-associated viruses (AAVs) carrying hSyn1 promotor-driven channelrhodopsin 2 (ChR2-eYFP) were injected into the premotor cortex to achieve neuronal expression of ChR2 (ref. ^23^). An optical cannula was then placed above the layer V neurons in the premotor cortex such that action potentials in ChR2-expressing neurons can be stimulated with blue light (Fig. 1b,c). Similar to our previous findings^4^, 20 Hz optogenetic stimulation of premotor circuit (motor planning area) triggers complex motor output in ChR2-expressing mice (circular walking behavior; Supplementary Video 1). During blue light stimulation, mice were given 5-ethynyl-2′-deoxyuridine (EdU) to identify dividing cells. Three hours after optogenetic stimulation, proliferating OPCs (EdU^+^/PDGFRα^+^ cells) were measured in frontal white matter projections (cingulum), which contain the axons of the stimulated neurons (Fig. 1c,d).

**Extended Data Fig. 1:**
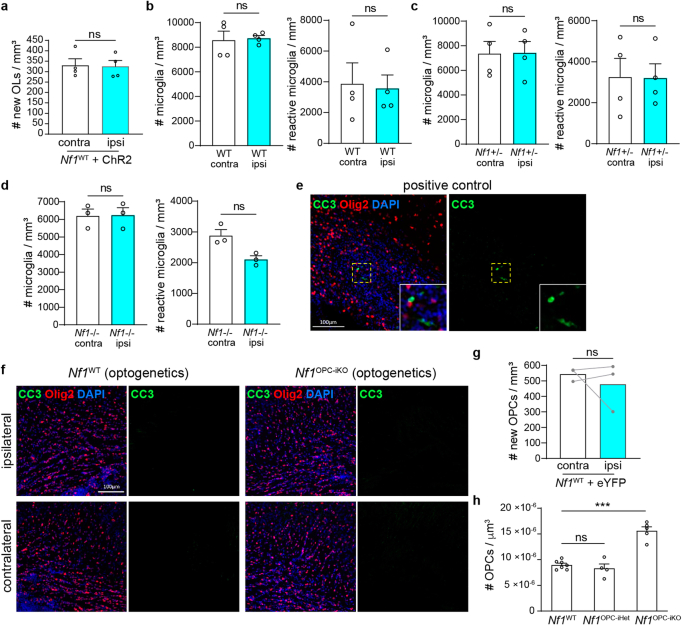
Optogenetic experiment controls. **(a)** Immunohistochemistry revealed no change in the density of new oligodendrocytes (EdU^+^/ASPA^+^) between the ipsilateral (blue, ipsi) and the contralateral (white, contra) side, during the 3-hour of the optogenetics experiment. Unpaired t test with Welch’s correction; N = 4 in each group. **(b-d)** Immunohistochemistry of the cingulum revealed no change in the density of microglia (Iba1^+^ cells) or reactive microglia (CD68^+^/Iba1^+^ cells) between the ipsilateral (blue, ipsi) and the contralateral (white, contra) side of *Nf1*^WT^ (WT, N = 4), *Nf1*^OPC-iHet^ (*Nf1*^+/-^, N = 4) and *Nf1*^OPC-iKO^ (*Nf1*^-/-^, N = 3) mice. Paired t test. **(e)** Cleaved caspase-3 (CC3) immunohistochemistry in the positive control (cuprizone-induced demyelination) showed staining of CC3^+^ (green) cells. Scale bar, 100 µm. **(f)** Immunohistochemistry of the cingulum from *Nf1*^WT^ and *Nf1*^OPC-iKO^ mice exposed to optogenetic stimulation revealed few CC3^+^ cells. Scale bar, 100 µm. **(g)** *Nf1*^WT^ mice infected with AAV carrying eYFP did not show changes in the density of new OPCs (EdU^+^/PDGFRα^+^) on the ipsilateral (blue, ipsi) side, relative to the contralateral (white, contra) side, of the brain. Mann-Whitney test; N = 3 in each group. **(h)** The density of total OPCs (PDGFRα^+^) in the contralateral side of optogenetically manipulated *Nf1*^OPC-iKO^ (N = 5), but not *Nf1*^OPC-iHet^ (N = 4), mice is increased relative to the *Nf1*^WT^ (N = 7) controls. Brown-Forsythe ANOVA test (F = 34.93) with Dunnett’s T3 multiple comparisons. ***, P = 0.0009. Data shown as mean ± SEM; each point = one mouse (a,b,c,d,g,h); two-sided; ns, not significant (P > 0.05). Source data

As expected for *Nf1*^WT^ mice^4^, optogenetically induced neuronal activity increased OPC proliferation ipsilateral to the site of optogenetic neuronal stimulation relative to the contralateral, nonstimulated side of the same mouse (Fig. 1e). During the 3 h following stimulation, newly proliferating OPCs have not yet differentiated into oligodendrocytes (Extended Data Fig. 1a); no microglial reactivity (Extended Data Fig. 1b–d) and few apoptotic cells (Extended Data Fig. 1e,f) were observed. As a control for surgical manipulation and blue light exposure, we expressed eYFP instead of ChR2-eYFP in *Nf1*^WT^ mice and observed no change in OPC proliferation following blue light delivery (Extended Data Fig. 1g), indicating that the increase in *Nf1*^WT^ OPC proliferation (Fig. 1e) results from optogenetic ChR2 activation of cortical projection neurons and is consistent with our previous findings^4^.

**Extended Data Fig. 2:**
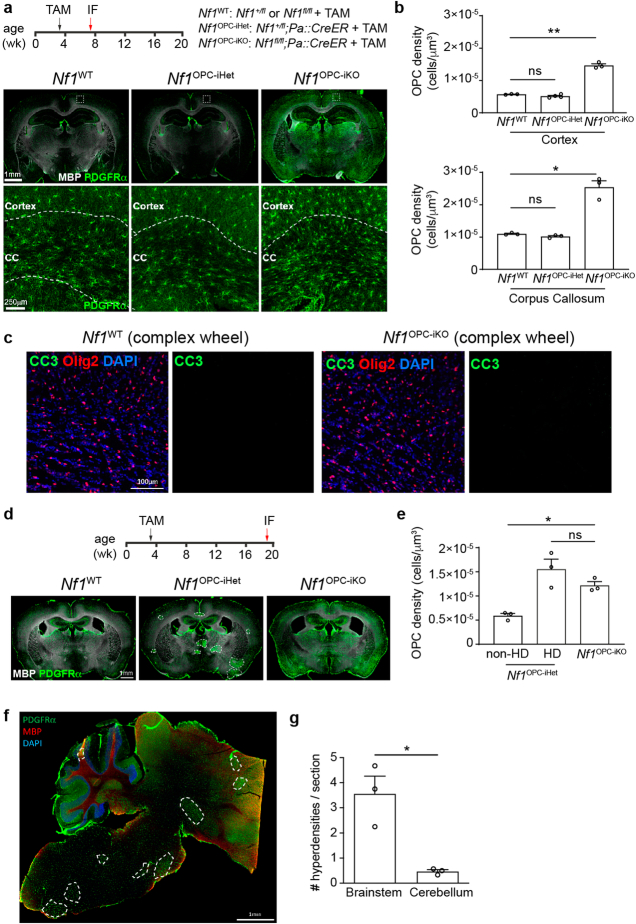
OPC-specific *Nf1* biallelic inactivation leads to global OPC hyperdensity, whereas *Nf1* monoallelic inactivation leads to focal OPC hyperdensities. **(a**, **b)** *Nf1* monoallelic and biallelic inactivation was induced at 3 weeks of age and mouse brains analyzed 4 weeks later by immunofluorescence (IF). Immunohistochemistry revealed increased OPC (PDGFRα^+^ cells) density in the brains of *Nf1*^OPC-iKO^ (N = 3), but not *Nf1*^OPC-iHet^ (N = 3 [corpus callosum], 4 [cortex]), mice relative to the *Nf1*^WT^ controls (N = 3). Brown-Forsythe ANOVA test (F = 55.06 [corpus callosum], 250.9 [cortex]) with Dunnett’s T3 multiple comparisons; *, P = 0.0299; **, P = 0.0056; Scale bars, 1 mm (top), 250 µm (bottom). **(c)** Immunohistochemistry of the cingulum from *Nf1*^WT^ and *Nf1*^OPC-iKO^ mice exposed to complex wheel testing revealed few CC3^+^ cells. Scale bar, 100 µm. **(d-e)** *Nf1* monoallelic and biallelic inactivation was induced at 3 weeks of age, and brains analyzed 16 weeks later. PDGFRα immunohistochemistry (green) revealed globally increased OPC density in the brains of *Nf1*^OPC-iKO^ mice, and focal OPC hyperdensities (dash lines) in *Nf1*^OPC-iHet^ mice. N = 3 in each group; Brown-Forsythe ANOVA test (F = 13.82) with Dunnett’s T3 multiple comparisons; *, P = 0.0115; Scale bar, 1 mm. **(f)** Sagittal sections of *Nf1*^+/-^ mouse brains were immunolabeled with PDGFRα (green) and MBP (red) antibodies. Scale bar, 1 mm. Dashed shapes indicate the focal OPC hyperdensities. **(g)** The number of focal OPC hyperdensities detected in each brain section from 16-week old *Nf1*^+/-^ mice. N = 3 per group. Welch’s t test; *, P = 0.0471. Data shown as mean ± SEM; each point = one mouse (b,e,g); two-sided; ns, not significant (P > 0.05). Source data

In striking contrast, optogenetically induced neuronal activity did not increase OPC proliferation in OPC-specific *Nf1*^OPC-iHet^ or *Nf1*^OPC-iKO^ mice (Fig. 1e), demonstrating that both monoallelic and biallelic *Nf1* inactivation abrogates the OPC proliferative response to neuronal activity. Notably, the overall density of proliferating OPCs is greater in *Nf1*^OPC-iKO^ mice (Fig. 1e) due to a generalized increase in total OPC density in *Nf1*^OPC-iKO^, relative to *Nf1*^WT^ and *Nf1*^OPC-iHet^, mouse brains (Extended Data Figs. 1h and 2a,b). This biallelic *Nf1* inactivation-induced increase in OPC density is consistent with previous findings demonstrating that *Nf1*-null OPCs exhibit increased proliferation^22^. Collectively, our findings reveal that *Nf1*-mutant OPCs lack the expected adaptive proliferative response to neuronal activity, and support a causative role for *Nf1* in regulating the homeostatic density of OPCs, which is strictly maintained in the healthy brain^24^.

## Deficiency in experience-regulated oligodendrogenesis

**Fig. 2:**
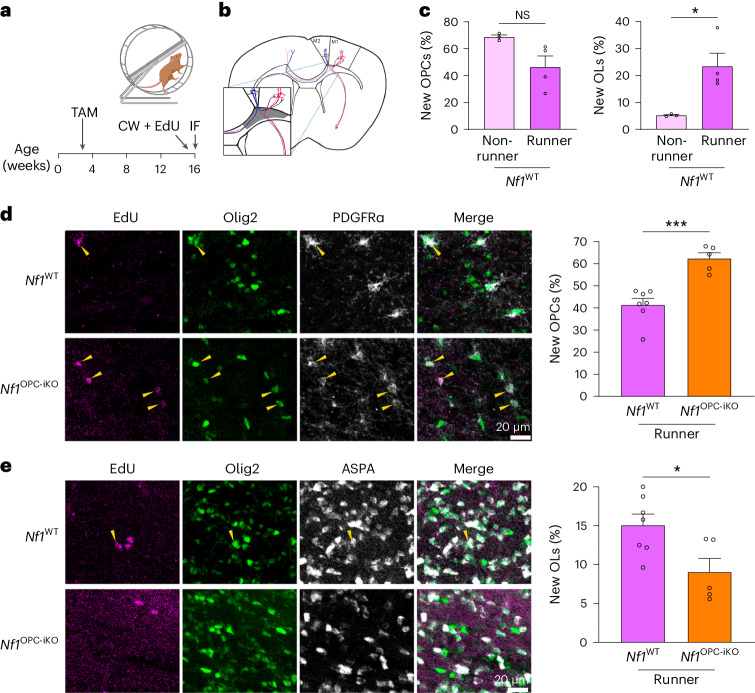
*Nf1*-deficient OPCs exhibit impaired experience-induced oligodendrogenesis. **a**, Experimental design. TAM, tamoxifen; CW, complex wheel; IF, immunofluorecence. **b**, OPC dynamics in cingulum (gray, inset) were analyzed by IF. **c**, Immunohistochemistry in *Nf1*^WT^ CW runners (*N* = 4) revealed no change in the percentage of new OPCs (number of EdU^+^/PDGFRα^+^ cells divided by EdU^+^ cells) and increased percentage of new oligodendrocytes (OLs, EdU^+^/ASPA^+^ divided by EdU^+^ cells), relative to *Nf1*^WT^ nonrunners (*N* = 3). *P = 0.0323. **d**, Immunohistochemistry revealed an increased percentage of new OPCs in *Nf1*^OPC-iKO^ mice (*N* = 5) relative to *Nf1*^WT^ (*N* = 7) mice at the end of CW test. ***P = 0.0003. Scale bars, 20 µm. **e**, Immunohistochemistry revealed a decreased percentage of new OLs in *Nf1*^OPC-iKO^ mice (*N* = 5) relative to *Nf1*^WT^ (*N* = 7) mice at the end of the CW test. *P = 0.0258. Scale bars, 20 µm. Pα, PDGFRα; unpaired *t*-test with Welch’s correction (**c**–**e**). Data shown as mean ± s.e.m.; each point represents one mouse (**c**–**e**); two-sided; NS, not significant (*P* > 0.05). Source data

Since *Nf1* mutation leads to OPC dysregulation, we next asked whether *Nf1* inactivation impairs OPC responses to neuronal activity in the context of motor learning as measured using the complex wheel test. The complex wheel has unevenly spaced rungs (Fig. 2a), requiring motor learning in order for the mouse to remain on the wheel. As mice learn, they run increasingly faster for the duration of the observation period^7^. This motor skill learning task induces OPC proliferation and the generation of new oligodendrocytes, and this activity-regulated oligodendrogenesis is necessary for complex wheel motor learning^7,8^.

Since loss of either one or both *Nf1* alleles in OPCs abrogates activity-regulated OPC proliferation (Fig. 1e), we hypothesized that *Nf1* mutation might also impair experience- and activity-regulated oligodendrogenesis. To evaluate this possibility, *Nf1*^WT^ and *Nf1*^OPC-iKO^ mice were given EdU in their drinking water during the complex wheel test to trace OPC proliferation and differentiation (Fig. 2a). At the end of 7 days of complex wheel training, we analyzed the percentage of newly proliferated OPCs (EdU^+^/PDGFRα^+^ cells) and newly generated oligodendrocytes (OLs; EdU^+^/ASPA^+^ cells) in the cingulum, where dense axonal tracks of the motor cortex reside (Fig. 2b). Consistent with previous studies^7,8^, *Nf1*^WT^ mice following complex wheel training harbor increased numbers of new oligodendrocytes without any change in EdU-labeled OPC content at this time point (Fig. 2c), indicating that in healthy mice this motor learning experience increases oligodendrogenesis in the cingulum and that by this time point OPCs that proliferated in response to the motor learning paradigm have differentiated into oligodendrocytes. In striking contrast, at the end of the test, mice with *Nf1*-null OPCs exhibit increased numbers of EdU-labeled OPCs, consistent with the overall increase in proliferating OPCs, and fewer new oligodendrocytes relative to their WT counterparts (Fig. 2d,e). No apoptosis (Extended Data Fig. 2c) after complex wheel training was detected at the time point examined to account for the observed reduction in new oligodendrocytes generated. Taken together, these data support a critical role for the *Nf1* gene in OPC differentiation and demonstrate a deficit in experience-dependent oligodendrogenesis in *Nf1*-null OPCs.

## *Nf1* loss generates OPC hyperdensities via PI3K/AKT activity

**Fig. 3:**
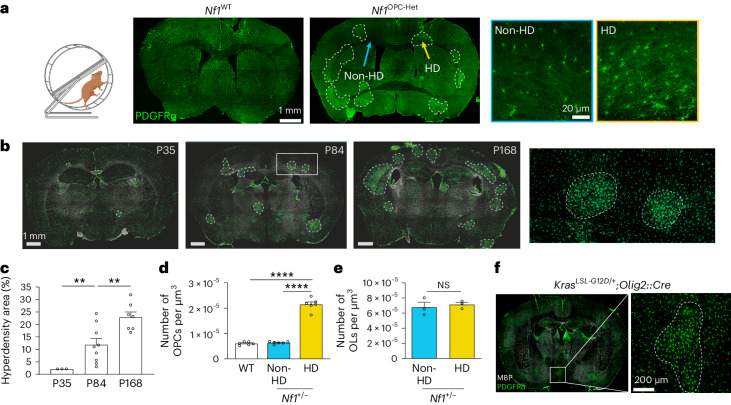
Monoallelic *Nf1* inactivation generates focal OPC hyperdensities with defective experience-dependent oligodendrogenesis. **a**, Left: experimental design. CW, complex wheel. Middle: immunohistochemistry of PDGFRα (green) revealed focal OPC hyperdensities (HD) in the *Nf1*^OPC-Het^ (*Nf1*^+/fl^;*Pdgfra::Cre*) mice. Scale bar, 1 mm. Right: representative non-HD (blue outline) and HD (yellow outline) OPCs (PDGFRα, green). Scale bar, 20 µm. **b**, PDGFRα immunohistochemistry (green) of *Nf1*^+/−^ mouse brains at P35, P84 and P168. Inset, two focal OPC hyperdensities and the surrounding nonhyperdensity regions. Scale bar, 1 mm. **c**, Quantification of **b** revealed an increased area of focal OPC hyperdensities in *Nf1*^+/−^ mouse brains at P35, P84 and P168. **P = 0.0092 (P35 versus P84), 0.0089 (P84 versus P168). *N* = 3, 9 and 7 (left to right). **d**, Quantification of OPC (PDGFRα^+^ cells) density in P35 mice revealed increased OPC density within the hyperdensity (HD) areas, relative to nonhyperdensity (non-HD) areas, in *Nf1*^+/−^ mouse brains and WT mouse brains. *N* = 6 per group. ****P < 0.001. **e**, Quantification of OL (ASPA^+^ cells) density in the cingulum of P184 *Nf1*^+/−^ mice revealed no differences between hyperdensities (HD) and nonhyperdensity (non-HD) areas. *N* = 3 per group. Unpaired *t*-test with Welch’s correction. **f**, Immunohistochemistry of PDGFRα (green) revealed focal OPC hyperdensities in the brains of *Kras*^*LSL-G12D/+*^*;Olig2::Cre* mice. Scale bar, 200 µm. Brown–Forsythe ANOVA test with Dunnett’s T3 multiple comparisons (**c**, *F* = 20.57; **d**, *F* = 216). Data shown as mean ± s.e.m.; each point represents one mouse (**c**–**e**); two-sided; NS, not significant (*P* > 0.05). Source data

To determine whether monoallelic *Nf1* inactivation also leads to deficits in experience-dependent oligodendrogenesis, we next analyzed OPC dynamics in *Nf1*^OPC-Het^ (*Nf1*^*fl/+*^*;Pdgfra::Cre*) mice at the end of the 7-day complex wheel test. Interestingly, we observed focal areas containing increased OPC density (focal OPC hyperdensities) throughout the brains of *Nf1*^OPC-Het^ mice (Fig. 3a), a finding indicative of impaired control of OPC density^24^. These focal OPC hyperdensities were also present in *Nf1*^OPC-iHet^ mice (*Nf1*^*fl/+*^*;Pdgfra::Cre*^*ER*^, tamoxifen injected at P24) not subjected to the complex wheel testing (Extended Data Fig. 2d,e), suggesting that formation of focal OPC hyperdensities is not a motor learning-driven event. OPC density within the focal OPC hyperdensities of OPC-specific *Nf1-*heterozygous mice is similar to that observed globally in the brains of *Nf1*^OPC-iKO^ mice (Extended Data Fig. 2e). In addition to the forebrain, focal OPC hyperdensities were also found in the hindbrains of *Nf1*^+/−^ mice, more often seen in the brainstem than the cerebellum (Extended Data Fig. 2f,g). These findings raise the intriguing idea that these regions of OPC hyperdensity, in which the normal OPC territorial boundaries^24^ are not respected, represent areas in which OPCs have lost expression of the remaining functional *Nf1* allele (*Nf1*-null OPCs).

**Extended Data Fig. 3:**
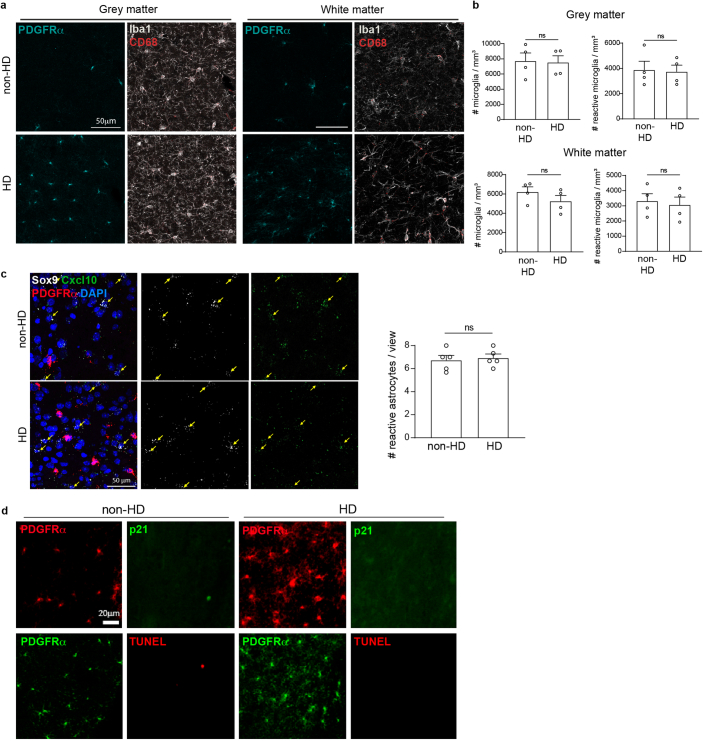
The focal OPC hyperdensities in the *Nf1*^+/-^ mouse brain lack obvious changes in other glial cells, senescence, or apoptosis. **(a**, **b)** Immunohistochemistry of *Nf1*^+/-^ mouse brains revealed no changes in the density of microglia (Iba1^+^ cells) or reactive microglia (CD68^+^/Iba1^+^ cells) in regions with focal OPC hyperdensities (HD) relative to those without (non-HD). Wilcoxon test (grey matter Iba1^+^ cells); Paired t test (other comparisons); Scale bars, 50 µm. N = 4 per group. **(c)** RNAscope revealed no changes in reactive astrocytes (*Cxcl10*^+^/*Sox9*^*+*^ cells) in areas with focal OPC hyperdensities relative to those without. N = 5 per group. Paired t test; Scale bar, 50 µm. **(d)** Immunohistochemistry revealed no senescent (p21^+^) or apoptotic (TUNEL^+^) cells in areas with OPC hyperdensities (HD), relative to those without (non-HD). Scale bar, 20 µm. Data are shown as mean ± SEM; each point = one mouse (b,c); two-sided; ns, not significant (P > 0.05). Source data

**Extended Data Fig. 4:**
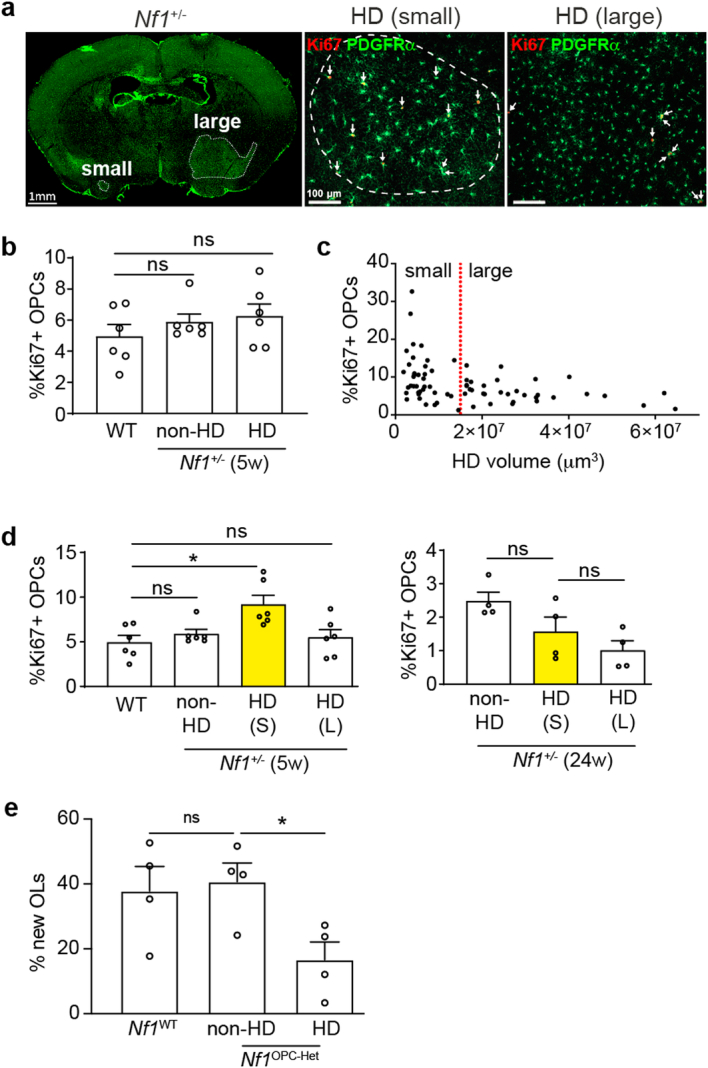
OPCs in small focal OPC hyperdensities show a temporary increase in proliferation. **(a)** Immunohistochemistry revealed that small and large focal OPC hyperdensities, with proliferating OPCs (Ki67^+^/PDGFRα^+^), are present at the same time in the brain of *Nf1*^+/-^ mice. Scale bars, 1 mm (whole brain), 100 µm (insets). **(b)** Overall, the percent of proliferating OPCs (the number of Ki67^+^/PDGFRα^+^ cells divided by the number of total PDGFRα^+^ cells) does not change between non-focal OPC hyperdensity- (non-HD, N = 6) and focal OPC hyperdensity-containing (HD, N = 6) areas of 5 week old *Nf1*^+/-^ mice, and is similar to the percent in WT mice (N = 6). Kruskal-Wallis test. **(c)** The focal OPC hyperdensities (HD) in *Nf1*^+/-^ mouse brains are divided into small and large size groups, using 1.5 × 10^7^ µm^3^ as an arbitrary cut-off. **(d)** Separating data in (b) based on the cut-off in (c) revealed that increased percent of proliferation was only observed in small (S) HD areas in 5-week-old *Nf1*^+/-^ mouse brains (N = 6 in each group), but not in small HD areas of 24-week-old *Nf1*^+/-^ mice (N = 4 in each group), or large (L) HD areas. Kruskal-Wallis test with Dunnett’s T3 multiple comparisons (5w); Brown-Forsythe ANOVA test (24w, F = 4.85) with Dunnett’s T3 multiple comparisons; *, P = 0.0224. **(e)** *Nf1*^WT^ and *Nf1*^OPC-Het^ mice were pulsed with EdU (40 mg/kg, i.p.) at P30-31. Immunohistochemistry performed at P37 revealed that focal OPC hyperdensities (HD) in the *Nf1*^OPC-Het^ mice exhibited reduced percentage of new OLs (the number of EdU^+^/Olig2^+^/PDGFRα^-^ cells divided by the number of EdU^+^ cells) relative to non-HD regions. N = 4 per group. Brown-Forsythe ANOVA (Dunnett); *, P = 0.0444. Data shown as mean ± SEM; each point = one mouse (b,d,e); two-sided; ns, not significant (P > 0.05). Source data

**Extended Data Fig. 5:**
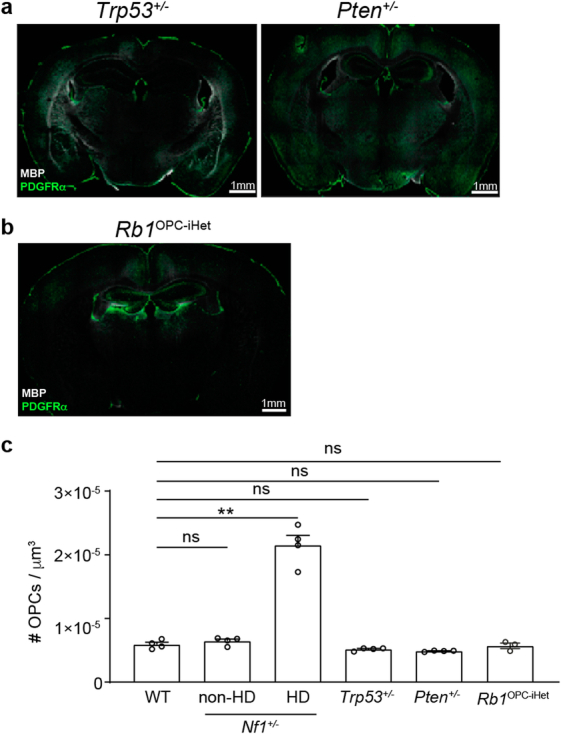
Focal OPC hyperdensities are not detected in mice harboring monoallelic inactivation of other tumor suppressor genes. **(a)** Immunohistochemistry revealed no focal OPC hyperdensities (HD) in *Trp53*^+/-^ or *Pten*^+/-^ mice. Scale bar, 1 mm. **(b)** No focal OPC hyperdensities were observed in *Rb1*^OPC-iHet^ (*Rb1*^*+/fl*^*;Pdgfra::Cre*^*ER*^ injected with tamoxifen) mice. Scale bar, 1 mm. **(c)** OPC density in *Trp53*^*+/-*^, *Pten*^*+/-*^, and *Rb1*^OPC-iHet^ mice is similar to WT mice. Brown-Forsythe ANOVA test (F = 91.3) with Dunnett’s T3 multiple comparisons. **, P = 0.0076. N = 4,4,4,4,4,3 (left to right). Data shown as mean ± SEM; each point = one mouse (c); two-sided; ns, not significant (P > 0.05). Source data

Within these focal OPC hyperdensities, no changes in the density of microglia (Iba1^+^ cells), reactive microglia (CD68^+^/Iba1^+^ cells) or reactive astrocytes (*Cxcl10*^+^/*Sox9*^+^ cells) were observed (Extended Data Fig. 3a–c). We also did not detect senescent cells (p21^+^ cells) or apoptotic cells (TUNEL^+^ cells) within these focal OPC hyperdensities (Extended Data Fig. 3d). The size of the focal OPC hyperdensities appears to increase with age (Fig. 3b–d). We found a transient increase in OPC proliferation in small focal OPC hyperdensities at 5 weeks, but not at 24 weeks, of age compared to regions with normal OPC density in the same brains (Extended Data Fig. 4a–d). Whereas the ability of OPCs to generate new oligodendrocytes (evidenced by EdU tracing) is reduced in the focal OPC hyperdensities relative to nonhyperdense areas (Extended Data Fig. 4e), the density of mature oligodendrocytes inside and outside of the focal OPC hyperdensities did not differ in adult mice (Fig. 3e). In contrast, mice harboring monoallelic inactivation of other tumor suppressor genes (for example, *Trp53*, *Pten* and *Rb1*) did not exhibit focal OPC hyperdensities (Extended Data Fig. 5), establishing this phenotype as unique to NF1.

**Extended Data Fig. 6:**
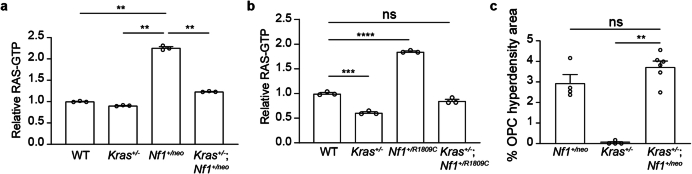
KRAS activity is not required for the development of focal OPC hyperdensities in heterozygous *Nf1*-mutant mice. **(a)** Heterozygous *Kras* loss rescues RAS hyperactivation in heterozygous *Nf1*-mutant mice. Brown-Forsythe ANOVA test (F = 1582) with Dunnett’s T3 multiple comparisons. **, P = 0.0012 (WT vs *Nf1*^+/neo^ mice), 0.001 (*Nf1*^+/neo^ vs *Kras*^+/-^ mice), 0.0018 (*Nf1*^+/neo^ vs *Kras*^+/-^;*Nf1*^+/neo^ mice). N = 3 per group. **(b)** *Kras* heterozygous loss rescues RAS hyperactivation in mice heterozygous for the R1809C *Nf1* gene mutation (1809; 5425 C > T)^35^. Brown-Forsythe ANOVA test (F = 610) with Dunnett’s T3 multiple comparisons. ***, P = 0.0003; ****, P < 0.0001. **(c)** The percentage of the forebrain covered by focal OPC hyperdensities revealed no difference between *Nf1*^+/neo^ and *Kras*^+/-^; *Nf1*^+/neo^ mice. Kruskal-Wallis test with Dunn’s multiple comparisons. **, P = 0.0071. N = 4,4,6 (left to right). Data shown as mean ± SEM; each point = one mouse (a-c); two-sided; ns, not significant (P > 0.05). Source data

As a GTPase-activating protein, one of the main functions of the *NF1* protein (neurofibromin) is to negatively regulate RAS activity. Using mice in which constitutively active KRAS is targeted to oligodendroglial lineage cells (*Kras*^*LSL-G12D*^*;Olig2::Cre*), KRAS hyperactivation phenocopies *Nf1* loss (Fig. 3f), indicating that increased KRAS activity is sufficient to induce focal OPC hyperdensities. To determine whether KRAS is necessary for inducing focal OPC hyperdensities in heterozygous *Nf1*-mutant mice, we engineered *Kras* haploinsufficiency in the *Nf1*-mutant mice. Whereas *Kras* haploinsufficiency normalizes *Nf1* mutation-induced RAS hyperactivation and does not induce OPC hyperdensities on its own (Extended Data Fig. 6a–c), *Kras* haploinsufficiency fails to reduce the size of the focal OPC hyperdensities in heterozygous *Nf1*-mutant mice (Extended Data Fig. 6c). Taken together, these findings suggest that oncogenic RAS hyperactivation in OPCs is sufficient to generate OPC hyperdensities but is not fully responsible for OPC hyperdensity formation in *Nf1*-mutant mice.

**Fig. 4:**
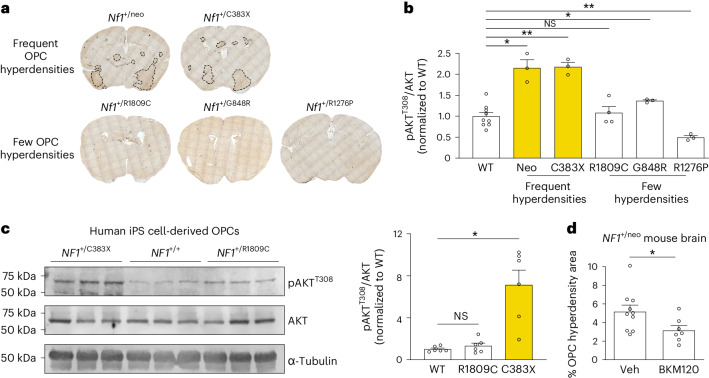
Heterozygous *Nf1*-mutant mouse brains contain focal OPC hyperdensities whose formation is *Nf1* mutation and PI3K dependent. **a**, Focal OPC hyperdensities (labeled by anti-PDGFRα antibody immunohistochemistry, brown) were detected in some, but not all, mice harboring NF1 patient-derived germline *Nf1* mutations. **b**, Immunoblotting revealed >2-fold increased AKT activity (phospho-AKT^T308^) in the brains of heterozygous *Nf1*-mutant mice with focal OPC hyperdensities in **a** relative to WT mice. *P = 0.042 (WT versus neo), 0.0138 (WT versus G848R); **P = 0.0015 (WT versus C383X), 0.0022 (WT versus R1276P). *N* = 9, 3, 3, 4, 3 and 3 (left to right). **c**, Immunoblotting of human iPS cell-derived OPCs heterozygous for *NF1*^+/C383X^, but not *NF1*^+/R1809C^, mutations have increased AKT activity (phospho-AKT^T308^) relative to WT OPCs. *P = 0.0135. *N* = 6 per group. **d**, Immunohistochemistry for the OPC marker PDGFRα revealed reduced area of focal OPC hyperdensities in the brains of *Nf1*^+/neo^ mice treated with NVP-BKM120 relative to mice treated with vehicle control (Veh). Unpaired *t*-test with Welch’s correction. *N* = 10,7 (left to right). *P = 0.0316. Brown–Forsythe ANOVA test with Dunnett’s T3 multiple comparisons (**b**, *F* = 29.16; **c**, *F* = 17.52). Data shown as mean ± s.e.m.; each point represents one mouse (**b**–**d**); two-sided; NS, not significant (*P* > 0.05). Source data

These findings indicating sufficiency but not necessity prompted us to further explore the causative etiology underlying OPC dysfunction in *Nf1*-mutant mice. First, we leveraged a collection of *Nf1*-mutant mouse strains harboring different heterozygous NF1 patient-derived germline *Nf1* gene mutations. Using this approach, we identified one line (*Nf1*^+/C383X^) that developed OPC hyperdensities similar to the *Nf1*-heterozygous mice engineered by inserting a neomycin cassette into exon 31 of the *Nf1* gene (*Nf1*^+/neo^), while three other lines (*Nf1*^+/R1809C^, *Nf1*^+/G848R^ and *Nf1*^+/R1276P^) did not exhibit OPC hyperdensities (Fig. 4a). In all of these NF1 model mouse strains, irrespective of OPC hyperdensity development, RAS activity is elevated^25–27^. Second, we examined PI3K-AKT signaling, which is also dysregulated in *Nf1*-mutant cells^28–30^. Examining AKT activity levels in brain lysates from WT and *Nf1*-mutant mice, we found that, relative to WT mice, AKT activity was increased by >2-fold in *Nf1*^+/neo^ and *Nf1*^+/C383X^ mice that form OPC hyperdensities but was largely not changed in *Nf1*^+/R1809C^, *Nf1*^+/G848R^ or *Nf1*^+/R1276P^ mice that harbor few or tiny OPC hyperdensities (Fig. 4b). Consistently, in OPCs derived from human induced pluripotent stem (iPS) cells with *NF1* mutations, only OPCs with the *NF1*^C383X^, but not the *NF1*^R1809C^, mutation exhibited elevated AKT activity relative to their WT controls (Fig. 4c). Third, we demonstrate that in vivo pharmacological PI3K/AKT inhibition using NVP-BKM120 reduced the OPC hyperdensity-affected area in the brains of *Nf1*^+/neo^ mice (Fig. 4d). Taken together, these data suggest that differential AKT activation underlies the development of OPC hyperdensities in *Nf1*-mutant mice.

## Impaired adaptive oligodendrogenesis in OPC hyperdensities

**Extended Data Fig. 7:**
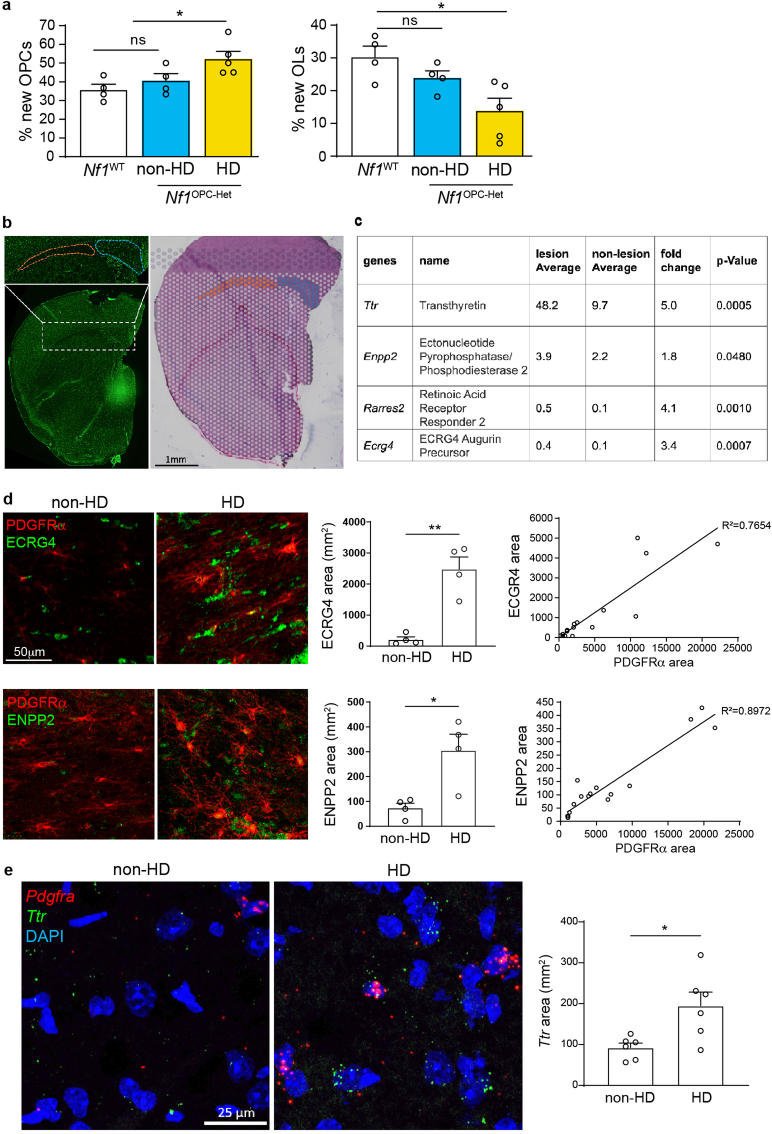
Spatial transcriptomic analysis of focal OPC hyperdensities. **(a)** Immunohistochemistry revealed an increased percent of new OPCs (left, the number of EdU^+^/PDGFRα^+^ cells divided by the number of EdU^+^ cells) and decreased percent of new OLs (right, the number of EdU^+^/ASPA^+^ cells divided by the number of EdU^+^ cells) in the HD areas of *Nf1*^OPC-Het^ mice (yellow, N = 5), relative to the non-HD areas of *Nf1*^OPC-Het^ mice (blue, N = 4) and *Nf1*^WT^ (white, N = 4) mice. *, P = 0.0255 (new OPC), 0.0272 (new OL). Brown-Forsythe ANOVA test with Dunnett’s T3 multiple comparisons (F = 5.812 [new OPC], 7.001[new OL]). **(b)** Representative image of a 24w-old *Nf1*^+/-^ mouse brain used for spatial transcriptomics. Left, PDGFRα (green) immunohistochemistry revealed focal OPC hyperdensities within the corpus callosum (blue, inset; orange represents non-hyperdensity area). Right, areas with focal OPC hyperdensities (blue) and those without (orange) are marked in the Loupe Browser for regional differential gene expression analyses. Scale bar, 1 mm. **(c)** Regional differential gene expression analyses of brains from four 24w-old *Nf1*^+/-^ mice revealed four genes increased in the focal OPC hyperdensities relative to the areas lacking hyperdensities in the corpus callosum. The p-value was adjusted using Benjamini-Hochberg correction for multiple tests in the Loupe Browser 6.0.0. **(d)** Immunofluorescence of *Nf1*^+/-^ brains revealed increased ECRG4 and ENPP2 expression within corpus callosum focal OPC hyperdensities (HD) relative to areas lacking hyperdensities (non-HD). The area of ECRG4/ENPP2 signal (green) positively correlates with the area of PDGFRα signal (red, linear regression). To measure ECRG4, ENPP2 and PDGFRα area, the threshold was adjusted to cover the fluorescence signal. The same threshold value was used for all images. Four images were collected for each animal, representing focal OPC hyperdensities and areas lacking hyperdensities. Unpaired t test with Welch’s correction. * P = 0.0334; ** P = 0.0086. N = 4 per group. Scale bar, 50 µm. **(e)** RNAscope of *Nf1*^+/-^ brains revealed increased *Ttr* expression within the focal OPC hyperdensities (HD) relative to areas lacking hyperdensities (non-HD). N = 6 per group. Both cortex and corpus callosum hyperdensities were included in the quantification. Unpaired t test with Welch’s correction. * P = 0.0262. Scale bar, 25 µm. Data shown as mean ± SEM; each point = one mouse (d,e); two-sided. ns, not significant (P > 0.05). Source data

To determine whether OPCs within the focal OPC hyperdensities of *Nf1*^OPC-Het^ mice exhibit the same activity-dependent defects seen in *Nf1*^OPC-iKO^ mice, we quantified new OPCs and new oligodendrocytes in the cingulum of *Nf1*^OPC-Het^ mice at the end of the complex wheel test (Fig. 3a). Whereas the OPCs in regions of normal OPC density behave similarly to *Nf1*^WT^ OPCs, OPCs within focal OPC hyperdensities display impaired oligodendrogenesis at the end of the complex wheel test (Extended Data Fig. 7a) similar to *Nf1*-null OPCs (Fig. 2d,e).

Since the experience-dependent oligodendrogenesis deficits within focal OPC hyperdensities of *Nf1*^OPC-Het^ mice are similar to those observed in *Nf1*-null OPCs, we postulated that these hyperdense OPCs probably exhibit *Nf1* loss of heterozygosity, resulting in reduced levels of *Nf1* mRNA and protein. Unfortunately, few reagents (probes and antibodies) currently exist to accurately quantitate *Nf1* RNA/protein by in situ hybridization or immunohistochemistry. We first evaluated multiple commercially available or laboratory-generated *Nf1* probes and antibodies without success. We next performed spatial transcriptomic analysis, which allows for evaluation of regional RNA expression, in OPC hyperdensities relative to areas lacking these hyperdensities in heterozygous *Nf1*-mutant mice (Extended Data Fig. 7b). While *Nf1* mRNA copy numbers in the brain were too low to evaluate differential expression, we detected increased expression of four genes (*Ttr*, *Enpp2*, *Rarres2* and *Ecrg4*) in the focal OPC hyperdensities relative to regions lacking focal OPC hyperdensities (Extended Data Fig. 7c). Many of the candidates we identified in the spatial transcriptomics analysis have been previously implicated in oligodendroglial lineage function. First, transthyretin (*Ttr*) is expressed by OPCs and has been reported to promote both OPC proliferation and differentiation^31^. Second, ECRG4 augurin precursor (ECRG4) is a hormone-like peptide that induces OPC senescence^32^. Third, ectonucleotide pyrophosphatase/phosphodiesterase 2 (ENPP2) induces the differentiation of OPCs from Olig2^+^ precursor cells in the developing zebrafish hindbrain^33^. Notably, ENPP6, another member of the ENPP family, serves as a marker of motor learning (complex wheel)-induced oligodendrogenesis^8^. Immunofluorescence (IF) and in situ hybridization validation of the spatial transcriptomic data revealed increased areas of Ecrg4 (IF), Enpp2 (IF) and *Ttr* (in situ hybridization) signal in regions of focal OPC hyperdensities within *Nf1*^+/−^ mouse brains relative to brain regions lacking focal OPC hyperdensities (Extended Data Fig. 7d,e).

Taken together, these findings demonstrate that *Nf1* loss leads to the development of focal OPC hyperdensities, within which OPCs exhibit defective experience-dependent oligodendrogenesis and dysregulated oligodendroglial gene expression relevant to OPC function.

## *Nf1*-mutant mice exhibit delayed oligodendroglial development

**Fig. 5:**
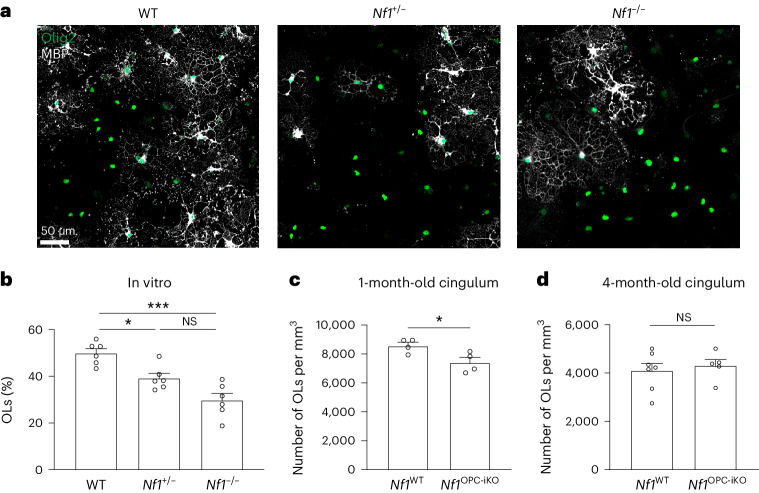
*Nf1* mutation inhibits OPC differentiation, and *Nf1*-mutant mice exhibit delayed oligodendroglial development. **a**,**b**, WT, *Nf1*^+/−^ and *Nf1*^−/−^ OPCs were cultured and induced for differentiation. Immunocytochemistry using MBP (oligodendrocyte marker) and Olig2 (oligodendroglial lineage marker) antibodies revealed a reduced percentage of oligodendrocytes (the number of MBP^+^ cells divided by the number of Olig2^+^ cells) in *Nf1*^+/−^ and *Nf1*^−/−^ OPCs, compared to the WT OPCs. Brown–Forsythe ANOVA test (*F* = 18.15) with Dunnett’s T3 multiple comparisons. *P = 0.0111; ***P = 0.0008. Scale bar, 50 µm. **c**, Immunohistochemistry performed in the cingulum of 1-month-old *Nf1*^WT^ and *Nf1*^OPC-iKO^ mice (tamoxifen injection at P28) revealed that *Nf1*^OPC-iKO^ mice had reduced density of oligodendrocytes (Olig2^+^/PDGFRα^−^ cells). *N* = 4 per group. *P = 0.0382. **d**, Immunohistochemistry in the cingulum of 4-month-old *Nf1*^WT^ (*N* = 7) and *Nf1*^OPC-iKO^ (*N* = 5) mice revealed similar oligodendrocyte (ASPA^+^ cells) density. Unpaired *t*-test with Welch’s correction (**c** and **d**). Data shown as mean ± s.e.m.; each point represents one mouse (**c** and **d**); two-sided; NS, not significant (*P* > 0.05). Source data

**Extended Data Fig. 8:**
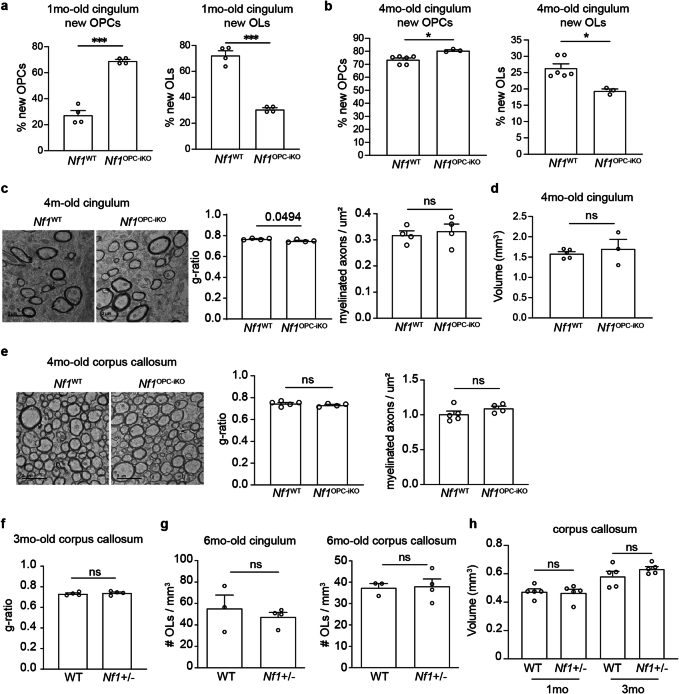
*Nf1*-mutant mice develop normal baseline oligodendrocyte density and myelination by young adulthood. **(a)** Immunohistochemistry performed in the cingulum of 1-month-old *Nf1*^WT^ and *Nf1*^OPC-iKO^ mice (tamoxifen injected at P28) revealed that *Nf1*^OPC-iKO^ mice had an increased percentage of new OPCs (the number of EdU^+^/Olig2^+^/PDGFRα^+^ cells divided by the number of EdU^+^/Olig2^+^ cells) and reduced percentage of new OLs (the number of EdU^+^/Olig2^+^/PDGFRα^-^ cells divided by the number of EdU^+^/Olig2^+^ cells) relative to *Nf1*^WT^ mice. N = 4 per group. ***, P = 0.0005. Welch’s test. **(b)** *Nf1*^WT^ and *Nf1*^OPC-iKO^ mice received tamoxifen at 13 weeks of age and pulsed with EdU in the drinking water for one week starting at 15 weeks of age. Immunohistochemistry performed at 16 weeks of age revealed that *Nf1*^OPC-iKO^ mice (N = 3) had an increased percentage of new OPCs (the number of EdU^+^/Olig2^+^/PDGFRα^+^ cells divided by the number of EdU^+^/Olig2^+^ cells) and reduced percentage of new OLs (the number of EdU^+^/Olig2^+^/PDGFRα^-^ cells divided by the number of EdU^+^/Olig2^+^ cells) relative to *Nf1*^WT^ mice (N = 6). Mann-Whitney test; *, P = 0.0238. **(c)** Transmission electron microscopy (TEM) analysis of myelin structure in the cingulum of 4-month-old *Nf1*^WT^ and *Nf1*^OPC-iKO^ mice revealed similar g-ratios (N = 4 per group) and densities of myelinated axons (N = 4 per group). Welch’s test. **(d)** Stereological analysis revealed similar cingulum volume between 4-month-old *Nf1*^WT^ (N = 5) and *Nf1*^OPC-iKO^ (N = 3) mice. Welch’s test. **(e)** TEM revealed similar g-ratios and density of myelinated axons in the corpus callosum of 4-month-old *Nf1*^WT^ (N = 5) and *Nf1*^OPC-iKO^ (N = 4) mice. Welch’s test. **(f)** TEM revealed similar g-ratios in the corpus callosum of 3-month-old WT and *Nf1*^+/-^ mice. N = 4 per group. Welch’s test. **(g)** Immunohistochemistry revealed a similar density of oligodendrocytes (ASPA^+^ cells) in the cingulum and corpus callosum of 6-month-old WT (N = 3) and *Nf1*^+/-^ (N = 4) mice. Welch’s test (cingulum). Mann-Whitney test (corpus callosum). **(h)** Stereological analysis revealed similar corpus callosum volume between WT and *Nf1*^+/-^ mice at 1-month and 3-month of age. N = 5 per group. Welch’s test. Data shown as mean ± SEM; each point = one mouse (a-h); two-sided; ns, not significant (P > 0.05). Scale bars, 2 µm. Source data

After observing that *Nf1* loss leads to defective experience-dependent oligodendrogenesis, we next asked whether baseline oligodendrogenesis is affected by *Nf1* loss in OPCs using EdU lineage tracing. In both 1-month-old and 4-month-old *Nf1*^OPC-iKO^ mice, we observed increased OPC proliferation and reduced differentiation relative to *Nf1*^WT^ mice, with smaller differences in the 4-month-old mice (Extended Data Fig. 8a,b). We next examined oligodendroglial lineage progression in OPCs isolated from *Nf1*^OPC-iKO^ and *Nf1*^WT^ pups. Using a standard OPC differentiation assay, *Nf1* loss (both monoallelic and biallelic) in OPCs decreases oligodendrogenesis in vitro (Fig. 5a,b).

Given the impaired OPC differentiation phenotype observed in *Nf1*-mutant OPCs, we quantified oligodendrocytes in developing and adult mice. At 1 month of age, *Nf1*^OPC-iKO^ mice exhibited reduced overall oligodendrocyte density compared to the *Nf1*^WT^ mice (Fig. 5c). This difference was no longer evident at 4 months of age (Fig. 5d). These findings indicate an impairment in developmental oligodendrogenesis that compensates by adulthood and suggests that the increased overall number of OPCs in *Nf1*^OPC-iKO^ mice may gradually compensate for their reduced capacity for oligodendrogenesis. Concordantly, by 4 months of age, we did not observe differences in myelination (number of myelinated axons and myelin sheath thickness) in the cingulum or corpus callosum of *Nf1*^OPC-iKO^ mice, as assessed by electron microscopy (Extended Data Fig. 8c–e), nor did we find differences in myelin or density of oligodendrocytes of adult *Nf1*^+/−^ mice (Extended Data Fig. 8f,g). The volume of the cingulum and corpus callosum was equivalent in *Nf1*-mutant and WT mice at all time points examined (Extended Data Fig. 8d,h).

Collectively, no gross deficits in baseline oligodendrocyte density or myelination were observed in *Nf1*-mutant mice at the age at which the complex wheel test was performed. We postulate that normal myelination is achieved by adulthood in *Nf1*-mutant mice, whereas activity-regulated oligodendrogenesis remains impaired. These data suggest that baseline abnormalities in OPC proliferation and differentiation contribute to the OPC/oligodendrocyte phenotype observed in *Nf1*-mutant mice following testing on the complex wheel and that failure of the activity-related response means that neuronal activity does not overcome this oligodendrogenesis deficit.

## *Nf1* loss in OPCs results in motor skill learning deficits

**Extended Data Fig. 9:**
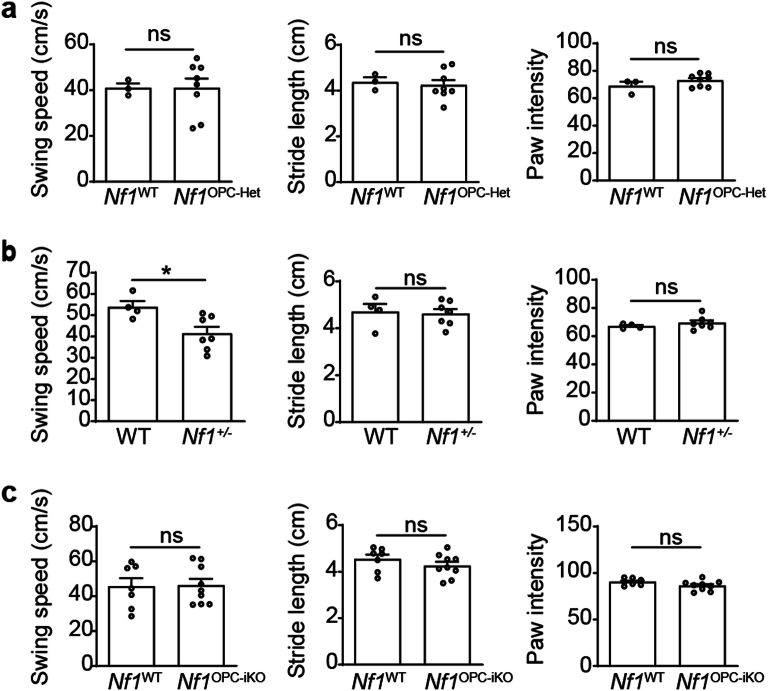
Gait analyses of *Nf1*-mutant mice. **(a)** CatWalk test revealed that 14–15 week old *Nf1*^OPC-Het^ (*Nf1*^+/fl^; *Pdgfra::Cre*) mice (N = 8) exhibit similar swing speeds, stride lengths, and paw intensities as *Nf1*^WT^ mice (N = 3). Unpaired t test with Welch’s correction (swing speed and stride length); Mann-Whitney test (paw intensity). **(b)** *Nf1*^+/-^ mice (N = 7, 14–15 week old) show reduced swing speed, but not stride length or paw intensity, relative to WT mice (N = 4). Unpaired t test with Welch’s correction; *, P = 0.0159. **(c)** *Nf1*^OPC-iKO^ mice (N = 9, 14–15 week old) show similar swing speed, stride length, and paw intensity as *Nf1*^WT^ mice (N = 7). Unpaired t test with Welch’s correction. Data shown as mean ± SEM; each point = one mouse (a,b,c); two-sided; ns, not significant (P > 0.05). Source data

Since *Nf1* loss leads to deficient experience-dependent oligodendrogenesis (Fig. 2d,e and Extended Data Fig. 7a), we next sought to determine whether OPC-specific *Nf1* loss causes impaired motor learning in adult mice. Before evaluating motor learning, we first assessed baseline motor function and found that the *Nf1*^OPC-Het^ mice lack abnormalities in overall motor function, including stride length, paw intensity and swing speed during normal gait (Extended Data Fig. 9a). This was important to confirm, as germline heterozygous *Nf1* loss (*Nf1*^*+/*^^−^ mice) results in motor function deficits (Extended Data Fig. 9b).

**Fig. 6:**
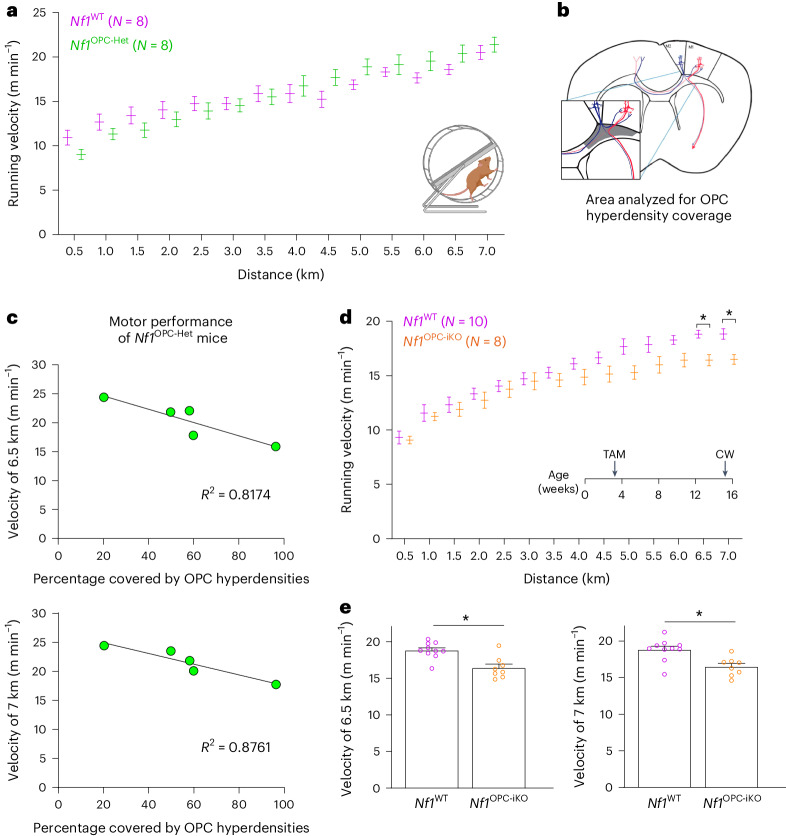
*Nf1* inactivation in OPCs results in motor learning deficits. **a**, *Nf1*^OPC-Het^ (*Nf1*^+/fl^;*Pdgfra::Cre*) mice (green, *N* = 8) showed a similar motor skill learning curve as *Nf1*^WT^ mice (magenta, *N* = 8). Two-way ANOVA with Bonferroni test. Comparisons between *Nf1*^WT^ and *Nf1*^OPC-iHet^ mice at each distance are not significantly different (*P* > 0.05). **b**, The shaded area represents the cingulum analyzed for the percent focal OPC hyperdensity coverage in **c**. **c**, The velocity of *Nf1*^OPC-Het^ mice at the end of the complex wheel test (6.5 and 7 km) is plotted against the percent area of cingulum covered by focal OPC hyperdensities. There is a negative correlation between the percent area covered by focal OPC hyperdensities and motor performance. Linear regression *R*^2^ = 0.8174 (6.5 km) and 0.8761 (7 km). **d**, Toward the end of the complex wheel test (6.5–7 km, **e**), *Nf1*^OPC-iKO^ mice (orange, *N* = 8) exhibited motor learning deficits relative to the *Nf1*^WT^ (magenta, *N* = 10) mice. **e**, The velocity of individual mice in **d** at 6.5 and 7 km. Two-way ANOVA with Bonferroni test. *P = 0.0332 (6.5 km) and 0.0372 (7 km). TAM, tamoxifen injection; CW, complex wheel. Data shown as mean ± s.e.m.; each point represents one mouse (**c** and **e**); two-sided (**a**, **d** and **e**).

Focusing on complex wheel performance (measured as running velocity) as a function of running distance, we found that all *Nf1*^OPC-Het^ mice, when grouped together, exhibit similar motor performance as their *Nf1*^WT^ counterparts (Fig. 6a). As the hyperdense OPC areas in *Nf1*^OPC-Het^ mice exhibit impaired experience-dependent oligodendrogenesis (Extended Data Fig. 7a), we correlated focal OPC hyperdensity size and location with mouse motor performance. *Nf1*^OPC-Het^ mice with more focal OPC hyperdensities within the cingulum exhibit lower running speeds at the end of the complex wheel test (6.5 and 7 km in running distance) (Fig. 6b,c). This finding indicates that the degree of focal OPC hyperdensities in subcortical motor projections inversely correlates with motor learning, consistent with the result described above demonstrating that experience-dependent oligodendrogenesis is impaired in these focal OPC hyperdensities (Extended Data Fig. 7a).

Given that OPCs within focal OPC hyperdensities of *Nf1*^OPC-Het^ mice behave like *Nf1*-null OPCs, we evaluated the hypothesis that biallelic *Nf1* inactivation in OPCs impairs motor learning. Baseline gait analyses demonstrated that *Nf1*^OPC-iKO^ mice do not differ from *Nf1*^WT^ mice with respect to swing speed, stride length or paw intensity (Extended Data Fig. 9c). During the complex wheel test, both *Nf1*^WT^ and *Nf1*^OPC-iKO^ mice increased their running velocity. However, *Nf1*^OPC-iKO^ mice exhibited slower velocities than *Nf1*^WT^ controls toward the end of the test (6.5- and 7-km running distance; Fig. 6d,e), illustrating impaired motor learning concordant with the impaired experience-dependent oligodendrogenesis shown above (Fig. 2d,e). Taken together, these results establish that OPC-specific *Nf1* biallelic inactivation leads to motor learning deficits.

## Discussion

In this study, we demonstrate that heterozygous *Nf1*-mutant and *Nf1*-null OPCs lack appropriate proliferative responses to neuronal activity and *Nf1*-deficient OPCs exhibit impairments in experience-induced oligodendrogenesis. In the brains of OPC-specific heterozygous *Nf1*-mutant mice, we identified focal OPC hyperdensities where OPCs within these hyperdensities exhibit impaired experience-induced oligodendrogenesis. Moreover, *Nf1* loss in OPCs alone is sufficient to cause motor learning deficits. Collectively, our findings provide a mechanistic connection between oligodendroglial plasticity and cognitive function in a neurogenetic disorder, raising several key points relevant to NF1 and OPC function.

First, neuronal activity is known to regulate OPC proliferation, oligodendrogenesis and myelination, which in turn mediates optimal circuit dynamics and several domains of neurological function, including attention, motor function, motor learning, spatial learning and memory consolidation^6–9^. Our group has previously demonstrated that optogenetically induced neuronal activity in the motor planning region (premotor, M2) increases OPC proliferation and oligodendrogenesis, leading to increased myelination of the cingulum and corpus callosum and improved motor function^4^. Using a similar in vivo optogenetic paradigm herein, we found that neither heterozygous *Nf1*-mutant nor *Nf1*-null OPCs exhibit proper neuronal activity-induced proliferation, suggesting that *Nf1* mutation disrupts the cellular/molecular mechanisms used by OPCs to sense and/or respond to activity-dependent signals. Since neuronal activity-regulated oligodendroglial responses support adaptive myelination to fine-tune circuit dynamics, the failure of *Nf1*-mutant OPCs to respond to neuronal activity implies that compromised oligodendroglial plasticity may partially contribute to the learning difficulties common in individuals with NF1.

In children with NF1, diffusion tensor imaging studies of white matter reveal differences in fractional anisotropy within the corpus callosum and cingulum compared to the control group; such differences were detected only during childhood (1–12 years of age) and not at adolescent ages^34^. These findings suggest delayed myelination during childhood that catches up later in adulthood. Supporting this hypothesis, we found reduced oligodendrocyte numbers in young (1-month-old) *Nf1*-mutant mice that normalize by adulthood. It is important to note that oligodendroglial cells also play myelin-independent roles, including axonal pruning^35^, synaptic pruning^36,37^, potassium buffering^38^ and antigen presentation^39^; *Nf1* mutations in OPCs could also affect these noncanonical oligodendroglial functions.

In the experience-dependent motor learning paradigm (complex wheel test), OPC-specific heterozygous *Nf1*-mutant mice show normal motor learning, whereas OPC-specific *Nf1*-null mice exhibit impaired motor learning. One plausible explanation for the motor learning difference between the heterozygous *Nf1*-mutant and *Nf1*-null conditions is that the complex wheel test-induced neuronal activity in the motor cortex (for example, both M1 and M2) is sufficient to induce some adaptive oligodendroglial changes in heterozygous *Nf1*-mutant but not in *Nf1*-null oligodendroglial cells. Notably, these two different models display baseline differences in their OPC populations: heterozygous *Nf1*-mutant mice show comparable OPC density to WT controls outside of hyperdense foci, whereas *Nf1*-null mice exhibit higher OPC density globally throughout their brains. It should be noted that not all newly generated oligodendrocytes derive from new OPC proliferation but by direct OPC differentiation without prior proliferation^8^. Additionally, existing oligodendrocytes can remodel myelin (for example, myelin sheath length) in response to neuronal activity^40,41^, underscoring the numerous mechanisms by which neuronal activity can modulate myelination; we have not assessed such potential myelin remodeling aspects of myelin plasticity in this study.

Activity-regulated oligodendroglial responses contribute to several cognitive functions, many of which are impaired in people with NF1, including attention, learning and memory^6–9^. Our findings show that mice with *Nf1*-mutant OPCs exhibit impaired responses to neuronal activity and consequent defective motor learning. While prior cell type-specific genetic mouse modeling studies demonstrated that *Nf1* loss in neurons causes spatial learning deficits^42^, it was unclear whether other neurological processes that oligodendroglial plasticity modulates (for example, attention, short-term memory and spatial learning) are similarly disrupted by *Nf1* mutation in OPCs. In this regard, we observed that larger focal OPC hyperdensities within the cingulum, which is critical for motor cortex function, correlate with poorer motor learning. These findings suggest a model where several neuronal circuits may be partially disrupted by the focal OPC hyperdensities, creating a potential threshold for the development or progression of neurological deficits.

The elucidation of an oligodendroglial-based mechanism for cognitive impairment in NF1 also suggests new potential therapeutic avenues focused on restoring adaptive oligodendroglial responses. Coupled with previous work demonstrating that a TrkB agonist, which induces oligodendrogenesis, rescues chemotherapy-induced impairment in oligodendroglial plasticity and cognitive function^6^ and that clemastine, which promotes oligodendrogenesis, rescues social isolation-induced deficits in adaptive myelination and social avoidance^43^, these findings support the concept that therapies that promote OPC differentiation could potentially be leveraged to treat cognitive deficits in individuals with NF1.

Second, the germline *NF1* mutation (monoallelic inactivation) affects all cell types in the body of individuals with this neurogenetic disorder; however, subsequent second-hit events (for example, *NF1* loss of heterozygosity) involving the one remaining functional *NF1* allele (biallelic inactivation) in specific cell types contribute to many NF1 clinical manifestations. Depending on the cell types affected, monoallelic and biallelic *NF1* inactivation can both be pathogenic. Using genetically engineered mice to model NF1, previous studies revealed that monoallelic inactivation of *Nf1* in inhibitory neurons leads to deficits in spatial learning^42^. In contrast, the NF1-associated corpus callosum enlargement was observed only with biallelic, but not monoallelic, *Nf1* inactivation in neural stem cells^44^. It is thus important to study the function of *NF1* in a cell type-specific manner and to investigate the effects of both monoallelic and biallelic *NF1* inactivation. In this study, we found that *Nf1*^OPC-iKO^ (biallelic *NF1* loss) mice exhibit impaired motor learning, while motor learning performance in *Nf1*^OPC-Het^ (monoallelic *NF1* loss) mice depends on the size and location of the focal OPC hyperdensities. Our findings also underscore the contribution of biallelic *Nf1* inactivation to more severe CNS deficits associated with NF1.

The discrete appearance of focal OPC hyperdensities in heterozygous *Nf1*-mutant mouse brains is reminiscent of the T2 hyperintensities (focal areas of signal intensity, FASI) detected on magnetic resonance imaging of children with NF1 (ref. ^45^). Prior histological analyses of the brains of three patients with FASI uncovered vacuolar changes suggestive of myelin disruption^46^. However, the cellular identity of FASI remains inconclusive so far. The OPC density and the experience-dependent oligodendrogenesis deficits of OPCs within focal OPC hyperdensities of heterozygous *Nf1*-mutant mice are similar to the behavior of *Nf1*-null OPCs, suggesting that OPCs within focal OPC hyperdensities probably exhibit *Nf1* loss of heterozygosity. In support of this idea, OPC-specific *Nf1* loss of heterozygosity induced in heterozygous *Nf1*-mutant mice by Cre-mediated chromosomal recombination (MADM) results in regional increases in OPC proliferation^22^.

Third, the finding that some, but not all, germline *Nf1* mutations result in the formation of OPC hyperdensities suggests differential effects of the mutation on oligodendroglial lineage biology. In this respect, there are *Nf1* mutation-specific effects, which are not accounted for by neurofibromin regulation of RAS, as all *Nf1* mutations examined lead to increased RAS activity. In contrast, the germline *Nf1* mutations associated with OPC hyperdensity formation result in increased PI3K/AKT signaling, suggesting additional functions of neurofibromin in maintaining PI3K/AKT homeostasis independent of RAS hyperactivation alone. Importantly, these observations dissociate OPC hyperdensity from optic glioma formation in that some strains that do not generate focal OPC abnormalities still form optic gliomas (R1278X)^47^. Future investigation will be required to define the mechanism by which germline *Nf1* mutations differentially regulate PI3K/AKT activation in OPCs.

Last, NF1 is both a neurological disorder and a cancer predisposition syndrome. It is conceivable that the focal OPC hyperdensities represent preneoplastic regions at risk of transforming into gliomas. To this end, inactivating both *Nf1* and *Trp53* transforms OPCs into high-grade gliomas^14^. It is therefore possible that *Nf1* inactivation primes OPCs for neoplastic transformation by increasing proliferation and decreasing oligodendrogenesis, while *Trp53* inactivation is required to inhibit the senescence program in *Nf1*-null OPCs^22^ and facilitate gliomagenesis. Given that adult patients with NF1 have a higher chance of developing high-grade gliomas than observed in the general population^48,49^, it is likely that these focal OPC hyperdensities serve as a preneoplastic pool of glioma-initiating cells that transform into glioma when mutations in other glioma driver genes (for example, *Trp53*) co-occur.

## Conclusion

Oligodendroglial plasticity is critical for proper neurological function in the healthy brain, and we now demonstrate that adaptive OPC responses are disrupted by *NF1* mutations in the neurogenetic disorder NF1, which impairs oligodendroglial dynamics and results in motor learning deficits.

## Methods

### Mice

All mice were used in accordance with an approved Institutional Animal Care and Use Committee protocol at Stanford University and Washington University. All mice were maintained on a C57/BL6 background. Mice were housed with free access to water and food according to the university’s guidelines in 12-h light/12-h dark cycles. The housing rooms are kept at a set point of 20–26 °C, with humidity ranging from 30% to 70%. Littermates (4–24 weeks of age) were used without selection for sexes. No obvious sex-dependent effect was observed. *Nf1*^WT^ (*Nf1*^*fl/fl*^, *Nf1*^*fl/+*^ or *Nf1*^*+/+*^) and heterozygous *Nf1*-mutant (*Nf1*^*fl/mut*^ or *Nf1*^*+/mut*^) mice were generated previously^50–53^ and bred with *Pdgfra::Cre*^*ER*^ (Jackson Laboratory, 018280) or *Pdgfra::Cre* mice (Jackson Laboratory, 013148) to induce OPC-specific *Nf1* inactivation. *Nf1*^+/C383X^, *Nf1*^+/R1809C^, *Nf1*^+/G848R^ or *Nf1*^+/R1276P^ mice were generated previously^47,54,55^. *Kras*^*LSL-G12D*^ mice (courtesy of Dr. Laura Attardi) were bred with the *Olig2::Cre* mice (025567). *Trp53*^*+/*^^−^ (002101), *Pten*^*+/*^^−^ (42059) and *Kras*^*+/*^^−^ (008179) mice were purchased from Jackson Laboratory; *Rb1*^+/fl^ mice (courtesy of Dr. Julien Sage)^56^ were bred with the *Pdgfra::Cre*^*ER*^ mice. To induce Cre^ER^-mediated *Nf1* inactivation, mice were administered tamoxifen (Sigma-Aldrich T5648, 100 mg kg^−1^, intraperitoneally (i.p.)) for 4 consecutive days (optogenetic and complex wheel experiments) or 4-hydroxytamoxifen (Sigma-Aldrich H6278, 50 mg kg^−1^, i.p.) for 5 consecutive days (immunohistochemistry analyses in Extended Data Fig. 3). NVP-BKM120 (PI3K inhibitor; 50 mg kg^−1^ daily; Selleckchem S2247) was administered to P23 *Nf1*^+/neo^ mice by oral gavage for 14 days.

### Optogenetic stimulation

AAV-DJ-hSyn-hChR2(H134R)-eYFP (virus titer: 1.5 × 10^12^ vg ml^−1^) and AAV-DJ-hSyn-eYFP (virus titer: 2.2 × 10^12^ vg ml^−1^) were obtained from Stanford Gene Vector and Virus Core. One microliter of virus was unilaterally injected into the premotor cortex (from bregma anterior-posterior (AP), +1.00 mm; medial-lateral (ML), −0.5 mm; dorsal-ventral (DV), −0.7 mm) of 4-week-old mice under 1–4% isoflurane anesthesia on a stereotactic surgery rig. Optic cannula was placed over premotor cortex (from bregma AP, +1.00 mm; ML, −0.5 mm; DV, −0.5 mm) and secured with dental cement. Four weeks after the surgeries, animals were connected to a 473-nm diode-pumped solid-state laser system with a monofiber patch cord. To optogenetically stimulate the premotor cortex, pulses of blue light were administered at 20 Hz, 50 ms pulse length, with alternating 30-s-light 2-min recovery periods. ChR2-expressing, but not YFP-expressing, mice exhibited unidirectional circling behavior when the blue light was on. During stimulation sessions, light was administered for 30 min, and mice were perfused 3 h after the start of the stimulation session. To label the dividing cells, all mice were injected i.p. with EdU (40 mg kg^−1^) for all the stimulation sessions.

### Immunohistochemistry

Mice were anesthetized with 2.5% avertin in phosphate-buffered saline (PBS) and transcardially perfused with PBS. The brain was removed and placed into 4% paraformaldehyde for 16–24 h at 4 °C, followed by 30% sucrose in PBS. Coronal floating brain sections (40 μm) were obtained, blocked with 3% normal donkey serum in Tris-buffered saline (TBS) with 0.3% Triton X-100 for 30 min at room temperature. Primary antibody incubation (1% normal donkey serum in TBS with 0.3% Triton X-100) follows, either for 4 h at room temperature or overnight at 4 °C. The brain sections were then washed in TBS, followed by secondary antibody incubation (1% normal donkey serum in TBS with 0.3% Triton X-100) for 2–4 h at room temperature, then mounted with prolong gold (Invitrogen P36930).

Primary antibodies used: goat anti-PDGFRα (1:250–1:500, R&D AF1062), rabbit anti-Olig2 (1:500, Abcam ab109186), rabbit anti-Ki67 (1:500, Abcam ab15580), rat anti-MBP (1:250, Abcam ab7349), chicken anti-GFP (1:500, Abcam Ab13970), rabbit anti-ASPA (1:250, EMD Millipore ABN1698), mouse anti-ECRG4 (1:250, OriGene TA320049), rabbit anti-ENPP2 (1:200, Invitrogen PA5-85221), rat anti-CD68 (1:200, Abcam ab53444), rabbit anti-Iba1 (1:1,000, Wako Chemicals 019-19741), goat anti-Olig2 (1:500, Novus Bio AF2418), rat anti-MBP (1:250, Abcam ab7349) and rabbit anti-cleaved caspase-3 (1:500, Cell Signaling Technology 9664). Secondary antibodies used: donkey anti-goat 488 (1:500, Jackson ImmunoResearch 705-545-147), donkey anti-goat 594 (1:500, Jackson Immunoresearch 705-585-003), donkey anti-goat 647 (1:500, Jackson ImmunoResearch 705-605-147), donkey anti-rabbit 594 (1:500, Jackson ImmunoResearch 711-585-152), donkey anti-rabbit 647 (1:500, Jackson ImmunoResearch 711-605-152), donkey anti-rabbit 488 (1:500, Jackson ImmunoResearch 711-545-152), donkey anti-chicken 488 (1:500, Jackson ImmunoResearch 703-545-155) and donkey anti-rat 647 (1:500, Jackson ImmunoResearch 712-605-150). Images were taken using a Zeiss Axio Imager M2, a Zeiss LSM 700 or a Zeiss 980 scanning confocal microscope and quantified using ImageJ (v 2.0.0).

### Fluorescence in situ hybridization

To obtain fresh frozen brain samples for RNAscope, mice were intracardially perfused with PBS. The brains were embedded and frozen in the optimal cutting temperature (OCT) compound (Tissue-Tek). The fresh frozen brains were sectioned into 12-µm slices, which were attached to Tissue Path Superfrost Plus Gold Slides (Fisher Scientific 15-188-48). The slides were stored at −20 °C for 1 h to dry the brain slices. Then, the slides were stored at −80 °C until use. Fluorescence in situ hybridization was performed using the RNAscope Fluorescent Multiplex Reagent kit v2-Mm (ACDBio 323100) in accordance with the manufacturer’s instructions. To visualize amplified RNA signals, Opal 520, 570 and 690 reagents (Akoya Biosciences FP1487001KT, FP1488001KT and FP1497001KT) were used. Then, the brain slices were mounted with prolong gold (Invitrogen P36930). Probes used were Mm *Pdgfra*-C2/-C3 (ACDBio 480661-C2/480661-C3), Mm-*Ttr* (ACDbio 424171), Mm-*Cxcl10*-C2 (ACDbio 408921-C2) and Mm-*Sox9* (ACDbio 401051). Images were taken using a Zeiss 800 and Zeiss 980 confocal microscope and analyzed using Fiji (v 2.3.0/1.53q).

To measure *Ttr* area, the threshold was adjusted to *Ttr* fluorescence signal. To get consistent data, the same threshold value was used for all images. To quantify the number of reactive astrocytes, *Cxcl10* and *Sox9* double-positive cells were counted. Only the 4′,6-diamidino-2-phenylindole covered with over four dots of *Cxcl10* and *Sox9* signal was counted as a reactive astrocyte. For both *Ttr* and reactive astrocyte experiment, fewer than four *Pdgfra*^*+*^ cells or more than six *Pdgfra*^*+*^ cells in the region of interest were considered as nonhyperdensity or OPC hyperdensity areas, respectively.

### Complex wheel test

Mice (15 weeks of age) were individually housed with the complex wheel with water (supplemented with 0.2 mg ml^−1^ EdU) and food. The training sessions for the complex wheel test are conducted over 7 days. The animals have free access to the wheel, water and food during the entire session. Complex wheels with dimensions previously described were constructed from laser-cut acrylic and assembled in laser-cut acrylic housings^7^. Wheels were mounted on stainless-steel axles with polytetrafluoroethylene bearings. Rung arrangements were configured as previously described^7^. To measure wheel speed, infrared distance sensors were mounted above each wheel to identify the movement of individual rungs across the sensor path during the wheel’s rotation. Analog output from these sensors was logged along with timestamps using an Arduino. Up to six wheels were active simultaneously per experiment, and the speed of *Nf1*^WT^ mice does not differ among wheels, indicating similar performance of the wheels. Further hardware specifications are available upon request.

Logged data from the Arduino-based monitoring system were processed in Matlab. The rotation of individual wheel rungs across the sensor path (rung intercepts) was identified via Matlab’s ‘findpeaks’ function. The data were then filtered to periods of active wheel rotation, defined as periods where rung intercepts occurred at less than 2-s intervals. One full revolution of each wheel was identified by a series of rung intercepts equal to the total number of rungs per wheel. The cumulative distance traveled by each mouse over the total number of revolutions in each experiment was calculated using the circumference of each wheel. Using a sliding window of size equal to the number of rungs per wheel, wheel speed was estimated by dividing the wheel circumference over the total time elapsed within a given sliding window. These values were then initially smoothed by moving average over 100-revolution intervals. Subsequently, average velocities were calculated and reported over a defined distance interval, such that mouse-to-mouse comparisons were made on the basis of total distance traveled. The code for complex wheel test analyses is available at Zenodo (10.5281/zenodo.10864194).

### Transmission electron microscopy

Mice were killed and perfused with PBS followed by the Karnovsky’s fixative (2% glutaraldehyde and 4% paraformaldehyde in 0.1 M sodium cacodylate). The samples were kept in Karnovsky’s fixative for more than 3 weeks, then post-fixed in 2% osmium tetroxide (EMS 19100) for 2 h at room temperature, washed three times with ultrafiltered water and then en bloc stained 1% uranyl acetate (EMS 541-09-3) overnight at 4 °C. Samples were then dehydrated in graded ethanol (30%, 50%, 75% and 95%) for 15 min each at 4 °C; the samples were then allowed to equilibrate to room temperature and were rinsed in 100% ethanol two times, followed by propylene oxide (EMS 20401) for 15 min. Samples were infiltrated with EMbed-812 resin (EMS 14120) mixed 1:1 with propylene oxide for 2 h followed by 2:1 EMbed-812/propylene oxide for 2 h. The samples were then placed into EMbed-812 for 2 h and then placed into TAAB capsules filled with fresh resin, which were then placed into a 65 °C oven overnight. Sections were taken at 80 nm on a Leica Ultracut S (Leica) and mounted on 100 mesh Cu grids (EMS FCF100-Cu). Grids were contrast stained for 30 s in 3.5% uranyl acetate in 50% acetone followed by staining in 0.2% lead citrate for 2 min. Samples were imaged using a JEOL JEM-1400 transmission electron microscope at 120 kV, and images were collected using a Gatan Orius digital camera.

The g-ratio, defined as the axonal diameter in its short axis divided by the diameter of the entire fiber in the same axis (axonal diameter/axonal diameter + myelin sheath), was measured using ImageJ. A total of 54–209 myelinated axons were quantified for each animal from 2–10 ×6,000 transmission electron micrographs. The number of myelinated axons was quantified from 16–25 ×6,000 transmission electron micrographs.

### Human iPS cell-induced OPCs

Human iPS cells were CRISPR–Cas9-engineered to harbor patient-derived *NF1* mutations as previously described by the Washington University Genome Engineering and iPSC Core Facility (GEiC)^54^. Human iPS cells were differentiated into hiPSC-derived OPCs (iOPCs) as previously described^57^. Briefly, iPS cells were induced into embryoid bodies by seeding 40,000 iPS cells at the bottoms of ultralow cell attachment U-bottom 96-well plates, and incubating them for 5 days in neural induction medium (NIM) (Dulbecco’s modified Eagle medium (DMEM)/F12, 1% nonessential amino acids and 1× N-2 supplement). Subsequently, the embryoid bodies were transferred onto poly-l-ornithine/laminin-coated six-well plates and incubated for 11 days in NIM supplemented with 20 ng ml^−1^ bFGF (PeproTech) and 2 µg ml^−1^ heparin (STEMCELL Technologies); 3 days in NIM supplemented with 100 nM retinoic acid (Sigma-Aldrich); 7 days in NIM supplemented with 100 nM retinoic acid, 1 µM purmorphamine (STEMCELL Technologies) and 1 × B-27; and then 11 days in NIM supplemented with 10 ng ml^−1^ bFGF, 1 µM purmorphamine and 1 × B-27. For OPC maturation and maintenance, the OPC colonies were incubated for 120 additional days in glial induction medium (DMEM/F12, 1× N1 (Sigma-Aldrich), 1× B27, 60 ng ml^−1^ T3, 100 ng ml^−1^ biotin (Sigma-Aldrich) and 1 µM cAMP (PeproTech)) supplemented with 10 ng ml^−1^ PDGF-AA, 10 ng ml^−1^ IGF-1 and 10 ng ml^−1^ NT3.

### Mouse OPC culture and differentiation assay

P4-5 mouse pups were rapidly decapitated and brains were processed in Hibernate-A medium (Thermo Fisher Scientific, A12475-01). Resulting tissue was enzymatically disassociated in buffer containing HEPES–Hanks’ Balanced Salt Solution (HBSS) with DNase (Worthington Biochemical LS002007) and Liberase (Roche Applied Sciences 05401054001) at 37 °C on a rotator. Then, tissue mixture was triturated with a 1,000-μl tip and passed through a 100-μm cell strainer. OPCs were isolated using the CD140 (PDGFRα) Microbead kit (MACS, Miltenyi Biotec 130-101-502) according to the manufacturer’s instructions. A total of 30,000 cells were seeded per well, on laminin-coated (Thermo Fisher Scientific, 23017015) cover slips in a 24-well plate. OPC proliferation medium containing DMEM (Thermo Fisher Scientific, 11320082), GlutaMAX (Invitrogen, 35050-061), sodium pyruvate (Invitrogen, 11360070), MEM nonessential amino acids (Thermo Fisher Scientific, 11140076), antibiotic–antimytotic (Gibco), N21-MAX (R&D systems, AR012), trace elements B (Corning, 25-022-Cl), 5 mg ml^−1^ *N*-acetyl cysteine (Sigma-Aldrich, A9165), 10 ng ml^−1^ PDGF-AA (Shenandoah Biotechnology, 200-54), 10 ng ml^−1^ ciliary neurotrophic factor (PeproTech, 450-13) and 1 ng ml^−1^ NT3 (PeproTech, 450- 03) was used. OPCs were incubated in this proliferative medium for 3 days to allow for proliferation. After the cells were incubated in OPC proliferative medium for 3 days, they were switched to differentiation medium. This consists of OPC proliferation medium without the growth factors PDGF-AA and NT3. Half medium change was done every other day, and the differentiation assay continued for 6 days after the start of incubation with the differentiation medium. On the sixth day, cells were fixed with 4% paraformaldehyde for 20 min and incubated in HBSS until immunohistochemistry. All in vitro experiments were performed in triple wells (technical replicate) and independently replicated (biological replicates).

### Western blot

Snap-frozen whole mouse brains or iOPC pellets were lysed in 200 μl RIPA buffer (Fisher) supplemented with protease and phosphatase inhibitor cocktails (Cell Signaling Technology). Thirty micrograms of total protein of each sample was analyzed by western blot using antibodies, including rabbit anti-phospho-AKT^T308^ (Abcam ab38449, 1:500), rabbit anti-AKT (Cell Signaling Technology, 9272S, 1:1,000) and mouse anti-α–tubulin (Cell Signaling Technology, 3873S, 1:5,000). The membranes were imaged using a Li-Cor Odyssey Fc system and protein band intensities were analyzed using Li-Cor Image Studio Software (version 2.0). Relative expression of phospho-AKT^T308^ was calculated after normalization to total AKT levels and using α-tubulin as a loading control. A minimum of four independent brain samples or independently generated iOPC pellets were used for each genotype.

### Stereology

White matter volume was measured with the Cavalieri method by marking grid points over an area of interest and calculating with the Cavalieri Estimator in Stereo Investigator (v2023.1.2), as described previously^58^.

### Demyelination with lysolecithin

As the positive control for the cleaved caspase-3 immunostaining, 1 µl of lysolecithin (Sigma, 62963) was sterotaxically injected into cingulum (AP, +1 mm; ML, −1 mm; DV, −1.3 mm) of 4-week-old WT mice using Hamilton Neuros syringe (1701RN-65460-05) over 5 min with 0.2 µl min^−1^ flow rate. Animals were transcardially perfused 1 week later.

### CatWalk gait analysis

Mice (15 weeks of age) were tested on the CatWalk system (Noldus) before the complex wheel test. The test was performed as previously described^4^ in the dark with at least three successful runs recorded for each mouse. Data were analyzed with CatWalk XT 9.0 (Noldus).

### RAS activity assay

RAS activity assays (Cell Biolabs, STA-440) were performed on fresh flash-frozen hippocampi homogenized in the provided lysis buffer supplemented with aprotinin, leupeptin and phenylmethylsulfonyl fluoride. A total of 0.4–1 mg ml^−1^ of lysate was assayed per well, and the RAS activation enzyme-linked immunosorbent assay was performed following the manufacturer’s instructions. Each assay was performed using a minimum of three independently generated biological replicates. Data from these colorimetric assays were collected on a Bio-Rad iMark microplate reader and analyzed using MPM6 v6.3 (Bio-Rad Laboratories) software. In Extended Data Fig. 7a, one outlier was excluded from the *Kras*^+/^^−^ group (1.097) and one from the *Kras*^+/−^;*Nf1*^+/neo^ group (0.977) using the Grubbs test (*α* = 0.05).

### Spatial transcriptomics

For tissue optimization, ten serial sections of 10-μm thickness from a representative sample were subjected to bulk RNA extraction (Qiagen RNeasy Mini Kit, Qiagen) and RNA integrity number analysis (Bioanalyzer, Agilent). Seven serial sections of 10-µm thickness were collected on a Tissue Optimization slide (10x Genomics), hematoxylin and eosin (H&E) stained and subjected to permeabilization over a range of time points (3–30 min), followed by on-slide reverse transcription and fluorescent complementary DNA synthesis. Fluorescent cDNA footprint was imaged (Keyence BZ-X800, Keyence) and analyzed to obtain the appropriate permeabilization time for gene expression studies.

To analyze gene expression, 10-µm sections from fresh frozen OCT-embedded mouse brain tissue samples were collected on Visium Gene Expression Slides (10x Genomics). Sections were H&E stained and imaged (Keyence BZ-X800). Sections were permeabilized for 10 min, followed by reverse transcription, cDNA synthesis, cDNA amplification and next-generation sequencing library preparation as per the manufacturer’s protocol (10x Genomics).

For sequencing, indexed libraries were pooled and sequenced over NovaSeq 6000 (Illumina) to obtain a minimum of 50,000 reads per spot (250M reads per section). Raw sequencing reads were parsed through the Spaceranger analysis pipeline (10x Genomics) to generate the final readout. The analyses were performed using Loupe Browser (6.0.0).

### Statistics and reproducibility

Data analyses were performed using Prism GraphPad (v8.4.1). The normality of each group was determined by Shapiro–Wilk test. We did not assume equal variances. Comparisons between two normally distributed groups were analyzed using unpaired *t*-tests with Welch’s correction. Multiple comparisons among normally distributed groups were analyzed using Brown–Forsythe and Welch analysis of variance (ANOVA) tests with Dunnett’s T3 correction for multiple comparison. Kruskal–Wallis test with Dunn’s correction for multiple comparison was used for comparisons involving data that do not pass the Shapiro–Wilk test. Statistical tests used were indicated in the figure legends with *F* and *P* values. Statistical significance was set at *P* ≤ 0.05. All data are presented as mean values with standard error of mean (s.e.m.). The number of biological samples used in the in vivo experiments is indicated in the figure legends. Animals in each litter were randomly assigned to experimental groups. Data from multiple litters, each containing various genotypes, were pooled for analyses. Mouse ear tag and cage identifiers, with no additional information, were available to investigators who performed data collection and analyses. All in vitro tests were repeated at least three independent times with similar results. In motor learning tests, sample sizes were chosen on the basis of power calculations of pilot cohorts (80% power and significance level of 0.05). Sample sizes for other experiments were based on and similar to previously published studies^4–9^. Statistically significant outliers (based on Grubbs’ test) were excluded and indicated in figure legends.

### Materials availability

The reagents described herein are freely available and can be obtained by contacting the corresponding authors and with a standard materials transfer agreement.

### Reporting summary

Further information on research design is available in the Nature Portfolio Reporting Summary linked to this article.

## Online content

Any methods, additional references, Nature Portfolio reporting summaries, source data, extended data, supplementary information, acknowledgements, peer review information; details of author contributions and competing interests; and statements of data and code availability are available at 10.1038/s41593-024-01654-y.

### Supplementary information

Reporting Summary

Supplementary Video 1 Mice with neuronal ChR2 expression in the motor planning region were exposed to blue light (related to Fig. 1) and showed complex motor behavior.

### Source data

Source Data Figs. 1–5 Raw data.

Source Data Fig. 4 Unprocessed western blots.

Source Data Extended Data Fig./Tables 1–9 Raw data.

## Data Availability

Spatial transcriptomics data are deposited in GEO (accession number GSE263303). Source data are provided with this paper.

## Code availability

The Matlab code for complex wheel test analyses is available at Zenodo (10.5281/zenodo.10864194)^59^.
