# Supplementary material for: Nf1 mutation disrupts activity-dependent oligodendroglial plasticity and motor learning in mice
Source: Nat Neurosci. 2024 May 30;27(8):1555–64. doi: 10.1038/s41593-024-01654-y (PMC11303248; doi:10.1038/s41593-024-01654-y)
Supplement: Supplementary file 1 — Reporting Summary [file 41593_2024_1654_MOESM1_ESM.pdf]

nature portfolio

Corresponding author(s): Michelle Monje, MD, PhD, David H. Gutmann, MD, PhD, and Yuan Pan, PhD

Last updated by author(s): 03/21/2024

Reporting Summary

Nature Portfolio wishes to improve the reproducibility of the work that we publish. This form provides structure for consistency and transparency in reporting. For further information on Nature Portfolio policies, see our Editorial Policies and the Editorial Policy Checklist.

Statistics

For all statistical analyses, confirm that the following items are present in the figure legend, table legend, main text, or Methods section.

n/a	Confirmed	
☐	☒	The exact sample size (n) for each experimental group/condition, given as a discrete number and unit of measurement
☐	☒	A statement on whether measurements were taken from distinct samples or whether the same sample was measured repeatedly
☐	☒	The statistical test(s) used AND whether they are one- or two-sided<br>*Only common tests should be described solely by name; describe more complex techniques in the Methods section.*
☒	☐	A description of all covariates tested
☐	☒	A description of any assumptions or corrections, such as tests of normality and adjustment for multiple comparisons
☐	☒	A full description of the statistical parameters including central tendency (e.g. means) or other basic estimates (e.g. regression coefficient) AND variation (e.g. standard deviation) or associated estimates of uncertainty (e.g. confidence intervals)
☐	☒	For null hypothesis testing, the test statistic (e.g. F, t, r) with confidence intervals, effect sizes, degrees of freedom and P value noted<br>*Give P values as exact values whenever suitable.*
☒	☐	For Bayesian analysis, information on the choice of priors and Markov chain Monte Carlo settings
☒	☐	For hierarchical and complex designs, identification of the appropriate level for tests and full reporting of outcomes
☒	☐	Estimates of effect sizes (e.g. Cohen's d, Pearson's r), indicating how they were calculated

Our web collection on statistics for biologists contains articles on many of the points above.

Software and code

Policy information about availability of computer code

Data collection	Images were collected using Zeiss Axio Imager M2, a Zeiss LSM 700, a Zeiss 800, or a Zeiss 980 scanning confocal microscope. Gait characteristics were collected with CatWalk XT 9.0 (Noldus). Data for colorimetric assays were collected on a Bio-Rad iMark microplate reader. For spatial transcriptomics, indexed libraries were pooled and sequenced over NovaSeq 6000. Fluorescent cDNA footprint was imaged using Keyence BZX 800. Western blot membranes were imaged using a Li-Cor Odyssey Fc system. Samples processed for transmission electron microscopy were imaged using a JEOL JEM-1400 TEM at 120kV and images were collected using a Gatan Orius digital camera.
Data analysis	Immunohistochemistry images were analyzed with ImageJ (v 2.0.0) or Fiji (v 2.3.0/1.53q). Mouse running speed on complex wheel was analyzed using Matlab (R2018a). Data analyses were performed using Prism GraphPad (v8.4.1). Gait characteristics were analyzed with CatWalk XT 9.0 (Noldus). Data for colorimetric assays were analyzed using MPM6 v6.3 (Bio-Rad Laboratories) software. Raw RNA sequencing reads were parsed through the Spaceranger analysis pipeline (10x Genomics, Pleasanton, CA) to generate the final readout. Spatial transcriptomics analyses were performed using Loupe Browser 6.0.0. Western blot intensities were analyzed using Li-Cor Image Studio Software (version 2.0). White matter volume was measured with Stereo Investigator (v2023.1.2).

For manuscripts utilizing custom algorithms or software that are central to the research but not yet described in published literature, software must be made available to editors and reviewers. We strongly encourage code deposition in a community repository (e.g. GitHub). See the Nature Portfolio guidelines for submitting code & software for further information.

Data

Policy information about availability of data

All manuscripts must include a data availability statement. This statement should provide the following information, where applicable:

- Accession codes, unique identifiers, or web links for publicly available datasets
- A description of any restrictions on data availability
- For clinical datasets or third party data, please ensure that the statement adheres to our policy

Spatial transcriptomics data will be available via public databases upon manuscript publication.

Human research participants

Policy information about studies involving human research participants and Sex and Gender in Research.

Reporting on sex and gender	N/A
Population characteristics	N/A
Recruitment	N/A
Ethics oversight	N/A

Note that full information on the approval of the study protocol must also be provided in the manuscript.

Field-specific reporting

Please select the one below that is the best fit for your research. If you are not sure, read the appropriate sections before making your selection.

☒ Life sciences ☐ Behavioural & social sciences ☐ Ecological, evolutionary & environmental sciences

For a reference copy of the document with all sections, see nature.com/documents/nr-reporting-summary-flat.pdf

Life sciences study design

All studies must disclose on these points even when the disclosure is negative.

Sample size	In motor learning tests, sample sizes were chosen based on power calculations of pilot cohorts (80% power and significance level of 0.05). Sample sizes for other experiments were based on and similar to previously published studies (PMIDs: 29358753, 25324381, 24727982, 27455109, 31753579, 31122677).
Data exclusions	In the complex wheel test, mice that ran less than 7km in one week were excluded from analyses. One mouse that ran farther than 7km but showed sick behavior with weight loss and weakness was excluded.<br>In optogenetic experiments, mice that did not show locomotion in response to blue light stimulation were excluded. One significant outlier was removed in the Nf1+/- group (values of 2.33 and 2.48 in contralateral and ipsilateral sides, respetively), Whether significant outlier existed in one group was calculated based on Grubbs' test using GraphPad Outlier calculator.<br>One significant outlier was excluded from the WT ctrl group in the percent of new OLs after complex wheel test (value of 11.9)<br>In extended Data Fig. 6a, one outlier was excluded from the Kras+/- group (1.097) and one from the Kras+/-;Nf1+/neo group (0.977) using the Grubbs test (Alpha = 0.05).
Replication	The number of biological replicates (mice for in vivo experiment) is indicated in the figure legends, and was always three or greater. For each in vivo result, the experiment was performed in at least three litters of mice.
Randomization	Animals were randomized to experimental groups.
Blinding	Investigators were blinded to group allocation during data collection and analyses.

Reporting for specific materials, systems and methods

We require information from authors about some types of materials, experimental systems and methods used in many studies. Here, indicate whether each material, system or method listed is relevant to your study. If you are not sure if a list item applies to your research, read the appropriate section before selecting a response.

Materials & experimental systems

n/a	Involved in the study
☐	☒ Antibodies
☐	☒ Eukaryotic cell lines
☒	☐ Palaeontology and archaeology
☐	☒ Animals and other organisms
☒	☐ Clinical data
☒	☐ Dual use research of concern

Methods

n/a	Involved in the study
☒	☐ ChIP-seq
☒	☐ Flow cytometry
☒	☐ MRI-based neuroimaging

Antibodies

Antibodies used	Primary antibodies used: goat anti-PDGFRa (1:250-1:500, R&D AF1062), rabbit anti-Olig2 (1:500, Abcam ab109186), rabbit anti-Ki67 (1:500, Abcam ab15580), rat anti-MBP (1:250, Abcam ab7349), Chicken anti-GFP (1:500, Abcam Ab13970), Rabbit anti-ASPA (1:250, EMD Millipore ABN1698), rat anti-CD68 (1:200, Abcam ab53444), rabbit anti-Iba1 (1:1000, Wako Chemicals 019-19741), goat anti-Olig2 (1:500, Novus Bio AF2418), rat anti-MBP (1:250, Abcam ab7349), rabbit anti-cleaved caspase-3 (1:500, Cell Signaling Technology 9664), mouse anti-ECRG4 (1:250, Origene TA320049), rabbit anti-ENPP2 (1:200, Invitrogen PA5-85221), rabbit anti-phospho-AKT-T308 (Abcam #ab38449, 1:500), rabbit anti-AKT (Cell Signaling Technologies, #9272S, 1:1000), and mouse anti–tubulin (Cell Signaling Technologies, #3873S, 1:5000)<br>Secondary antibodies used: donkey anti-goat 488 (1:500, Jackson ImmunoResearch 705-545-147), donkey anti-goat 594 (1:500, Jackson Immunoresearch 705-585-003), donkey anti-goat 647 (1:500, Jackson Immunoresearch 705-605-147), donkey anti-rabbit 594 (1:500, Jackson ImmunoResearch 711-585-152), donkey anti-rabbit 647 (1:500, Jackson ImmunoResearch 711-605-152), donkey anti-rabbit 488 (1:500, Jackson ImmunoResearch 711-545-152), donkey anti-chicken 488 (1:500, Jackson Immunoresearch 703-545-155), and donkey anti-rat 647 (1:500, Jackson ImmunoResearch 712-605-150).
Validation	These primary antibodies were validated in mouse tissues or cells using IF/IHC with the expected intracellular localization and patterns of the expected cell types: anti-Ki67 (vendor), anti-Olig2 (vendor), anti-cleaved caspase-3 (vendor), anti-PDGFRa (PMID: 24727982), GFP (PMID: 34463618), ASPA (PMID: 33942715), anti-CD68 (PMID: 30528430), anti-Iba1 (PMID: 30528430), and MBP (PMID: 33217041). These primary antibodies were validated in human and mouse tissue/cell western blots with expected band size and changes in response to treatment: anti-phospho-AKT-T308 (vendor and PMID: 35165269), anti-AKT (vendor), and anti-tubulin (PMID: 36950124). The ECRG4 and ENPP2 staining IF in the mouse brain showed similar patterns as the spatial transcripomic data and previous publications using other antibody clones (PMID: 20404145, PMID: 29743582).<br>Sencondary antibody specificity were validated by the sencondary antibody only negative control in IF/ICC/WB.

Eukaryotic cell lines

Policy information about cell lines and Sex and Gender in Research

Cell line source(s)	The iPSCs were generated at the Washington university in st louis genome engineering and iPSC center. The mutations were CRISPR/Cas9- engineered on a control BJFF.6 male hiPSC line, originally reprogrammed by GEiC from commercially available male foreskin fibroblasts.
Authentication	Authentication of the iPSCs was performed by Immunocytochemical staining to ensure pluripotency
Mycoplasma contamination	The iPSCs were routinely tested for mycoplasma through commercially available kits and were mycoplasma-free
Commonly misidentified lines (See ICLAC register)	no commonly misidentified cell lines were used in the study

Animals and other research organisms

Policy information about studies involving animals; ARRIVE guidelines recommended for reporting animal research, and Sex and Gender in Research

Laboratory animals	All mice were maintained on a C57BL/6 background. mice were analysed at between 4-24 weeks of age. Nf1WT (Nf1fl/fl, Nf1fl/+ or Nf1+/+) and heterozygous Nf1-mutant (Nf1fl/mut or Nf1+/mut) mice were bred with Pdgfra::CreER (Jackson Laboratory, 018280) or Pdgfra::Cre mice (Jackson Laboratory, 013148) to induce OPC-specific Nf1 inactivation. Nf1+/C383X, Nf1+/R1809C, Nf1+/G848R or Nf1+/R1276P mice were used. KrasLSL-G12D mice (courtesy of Dr. Laura Attardi) were bred with the Olig2::Cre mice (025567). Trp53 +/- (002101), Pten+/- (42059), and Kras+/- (008179) mice were purchased from Jackson Laboratory; Rb1+/fl mice (courtesy of Dr. Julien Sage) were bred with the Pdgfra::CreER mice.
Wild animals	No wild animals were used.
Reporting on sex	Both male and female mice were used in this study.
Field-collected samples	No field-collected samples were used.
Ethics oversight	All mice were used in accordance with an approved Institutional Animal Care and Use Committee (IACUC) protocol at Stanford

Ethics oversight	University and Washington University. Mice were housed with free access to water and food according to the university's guidelines in 12 h light/12 h dark cycles. The housing rooms are kept at a set point of 20-26 °C, with humidity ranging from 30-70%.

Note that full information on the approval of the study protocol must also be provided in the manuscript.
