# Supplementary figures and images for: Nf1 mutation disrupts activity-dependent oligodendroglial plasticity and motor learning in mice
Source: Nat Neurosci. 2024 May 30;27(8):1555–64. doi: 10.1038/s41593-024-01654-y (PMC11303248; doi:10.1038/s41593-024-01654-y)

Fig. 4h

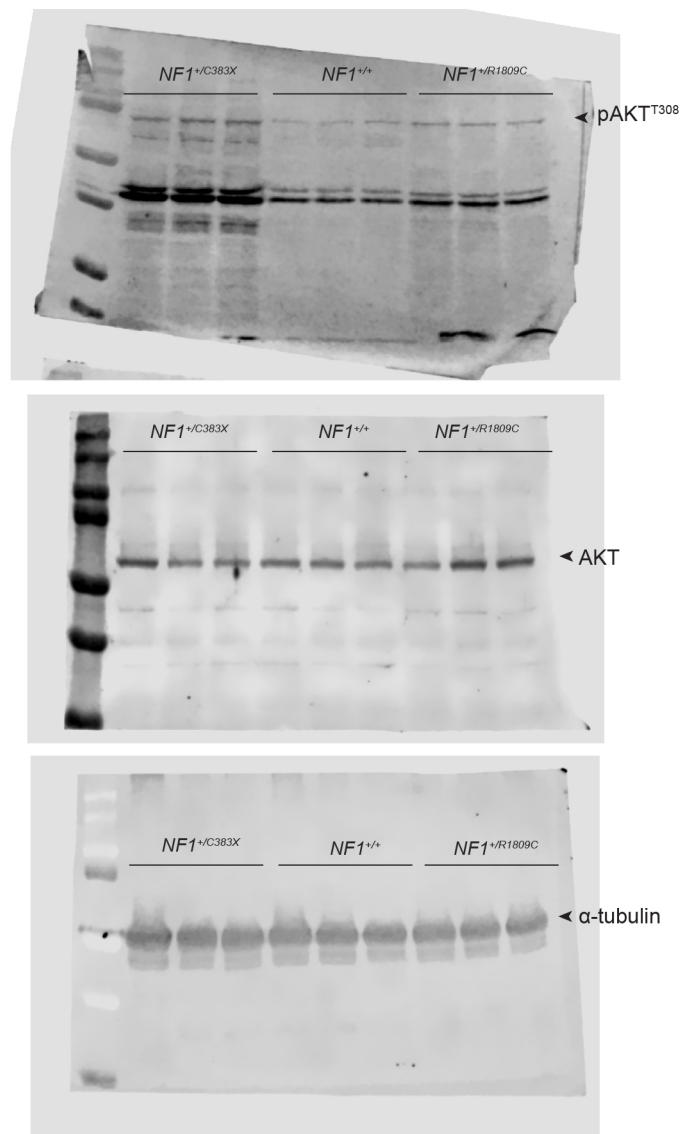

Supplement: Supplementary file 4 — Unprocessed western blots. [file 41593_2024_1654_MOESM4_ESM.pdf]
